# Integrating image-based phenotyping and GWAS to map resistance to spittlebug nymphs in interspecific *Urochloa* grasses

**DOI:** 10.1093/g3journal/jkag101

**Published:** 2026-04-27

**Authors:** Paula Espitia-Buitrago, Claudia Perea, Juan C Mejia-Medina, Luis M Hernández, Valheria Castiblanco, Camilla Ryan, José J De Vega, Rosa N Jauregui

**Affiliations:** Dept. Tropical Forages, International Center for Tropical Agriculture (CIAT), A.A. 6713, Km 17 recta Cali-Palmira, Palmira, Colombia; Dept. Tropical Forages, International Center for Tropical Agriculture (CIAT), A.A. 6713, Km 17 recta Cali-Palmira, Palmira, Colombia; Dept. Tropical Forages, International Center for Tropical Agriculture (CIAT), A.A. 6713, Km 17 recta Cali-Palmira, Palmira, Colombia; Dept. Tropical Forages, International Center for Tropical Agriculture (CIAT), A.A. 6713, Km 17 recta Cali-Palmira, Palmira, Colombia; Dept. Tropical Forages, International Center for Tropical Agriculture (CIAT), A.A. 6713, Km 17 recta Cali-Palmira, Palmira, Colombia; Earlham Institute, Norwich Bioscience Institutes, Norwich Research Park, Norwich NR4 7UZ, United Kingdom; Earlham Institute, Norwich Bioscience Institutes, Norwich Research Park, Norwich NR4 7UZ, United Kingdom; Dept. Tropical Forages, International Center for Tropical Agriculture (CIAT), A.A. 6713, Km 17 recta Cali-Palmira, Palmira, Colombia

**Keywords:** *Brachiaria*, *Urochloa*, grass, insect resistance, *Aeneolamia varia*, image-based phenotyping, GWAS, plant damage quantification

## Abstract

*Urochloa* grasses are among the most widely used forage grasses across the tropics. Spittlebugs (Hemiptera: Cercopidae) are major pests of tropical *Urochloa* (syn. *Brachiaria*) grass pastures, severely reducing forage productivity and quality. Understanding the genetic basis of host-plant resistance is essential for developing durable resistant cultivars. Here, we combined high-throughput image-based phenotyping and genome-wide association studies (GWAS) to dissect the genetic architecture of response to *Aeneolamia varia* nymphs in 339 interspecific F_1_ hybrids derived from crosses between resistant sexual and susceptible apomictic *Urochloa* parents. Digital image analysis using both unsupervised (DQU) and supervised (DTR) quantification pipelines enabled accurate estimation of plant damage, yielding moderate to high broad-sense heritability estimates (H^2^ = 0.49 to 0.66). In contrast, insect survival (NTS) exhibited low to moderate correlations with all damage traits and lower heritability estimates (H^2^ = 0.42). Using 57,051 high-quality SNPs aligned to the genome of the hybrid cultivar Basilisk, GWAS models identified 18 quantitative trait loci (QTLs) for plant damage traits, but none for insect survival (antibiosis). Six robust QTLs on chromosomes 1, 6, 7, 27, 29, and 36 were consistently detected across models and phenotyping methods, explaining up to 21.5% of phenotypic variance. Candidate gene analysis revealed proteins involved in hormone signaling, oxidative stress response, and cell wall modification, suggesting multifaceted plant-insect interaction mechanisms. These results provide a foundational set of molecular markers associated with spittlebug response in *Urochloa* grasses, useful for marker-assisted and genomic selection in the forage breeding program.

## Introduction

*Urochloa* grasses are among the most widely used forage grasses across the tropics. Spittlebugs (Hemiptera: Cercopidae) are a major pest of *Urochloa* P. Beauv. grasses (syn. *Brachiaria*) in tropical America, causing significant plant damage, particularly during the rainy season. Their infestations not only reduce forage production but also negatively impact nutritional quality, including crude protein content and in vitro digestible dry matter, resulting in reductions in livestock weight gain up to 74% per hectare (Congio et al. 2020). Given that pasture productivity directly impacts animal productivity in the dairy and beef production chains, host-plant resistance is the cornerstone of integrated pest management in *Urochloa* grass pasture systems.

Host-plant resistance is a particularly advantageous strategy against pests because it maintains pest populations below the economic injury threshold, it is fully compatible with other control methods, and it is inherently delivered through the seed, reducing long-term management costs (Cardona and Sotelo 2005). A resistant plant is defined as one that, under pest pressure, exhibits reduced damage as a result of its genetic makeup, with this resistance determined by various underlying mechanisms (i.e. traits that comprise the expression of resistance) and grouped into the resistance categories of antibiosis, antixenosis, and tolerance (Stout et al. 2024; Souza 2025). Antibiosis involves plant traits, e.g. metabolites acting as arthropod growth inhibitors or toxins, that negatively affect the biological development or survival of insect pests, and tolerance refers to the capacity of plants to withstand and compensate for herbivory without substantially compromising growth or yield, without affecting insect behavior or development (Mitchell et al. 2016; Huang et al. 2025; Souza 2025). Nonetheless, breeding for insect resistance remains difficult due to the high genetic polymorphism of insect species and being strongly shaped by environmental conditions and regulated by host–insect interactions (Priyadarshan 2019). Thus, identifying the resistance categories involved in genotypes’ responses to herbivory is crucial to diversifying the basis of resistance through diverse loci and, ultimately, building long-term resistance in breeding populations (Peterson et al. 2017; Pamidi et al. 2025).

Breeding programs for *Urochloa* aim to incorporate resistance traits present in diverse genotypes to develop improved cultivars. Notably, spittlebug resistance in *Urochloa* is both interspecific, where host genotype's response varies among spittlebug species, and intraspecific, where resistance depends on the insect's developmental stage (Cardona et al. 2010). To the best of our knowledge, the molecular mechanisms of resistance to spittlebugs have not been clearly elucidated in *Urochloa*. However, several factors have been proposed in past studies, including the production of volatile organic compounds (Silva et al. 2017, 2019), improved tolerance response with fertilization (Alvarenga et al. 2019), and activation of defense-related biochemical responses such as lipoxygenases, proteases, jasmonic acid (JA), and abscisic acid (ABA) (Barros et al. 2021).

Despite these findings, translating such resistance traits into practical outcomes through molecular breeding approaches in *Urochloa* grasses remains a significant challenge. The diverse reproductive modes, both apomictic and sexual, within intra- and interspecific crosses, a broad range of ploidy levels, and the outcrossing nature of *Urochloa* grasses, combined with limited understanding of chromosomal behavior during meiosis, unclear modes of inheritance, and high heterozygosity, significantly hinder crossing, evaluation, and selection activities (Ferreira et al. 2021). Despite these challenges, F_1_ families have been utilized to identify quantitative trait loci (QTLs) linked to key agronomic traits such as apomixis (Worthington et al. 2016, 2019), aluminum tolerance (Worthington et al. 2021), forage yield, and spittlebug antibiosis (Ferreira et al. 2019). The implementation of molecular markers in crossing, evaluation, and selection activities remains limited, with notable success primarily in marker-assisted selection of apomictic plants using markers p779/p780 (Worthington et al. 2016; Nitthaisong et al. 2019; da Costa Lima Moraes et al. 2023).

Previous QTL mapping studies in *Urochloa* have relied on the diploid *Urochloa ruziziensis* reference genome, which may not fully capture the genomic complexity of the polyploid interspecific hybrids commonly used in breeding programs. Recently, the allotetraploid reference genome of cv. Basilisk (CIAT 606) from the interspecific *Urochloa* complex has been released and found to be a hybrid of *Urochloa decumbens* and *Urochloa brizantha* (Ryan et al. 2025). It provides a more appropriate genomic framework for QTL analysis in polyploid breeding materials. Given the complexity of spittlebug resistance and the limited understanding of its genetic architecture, implementing this improved genomic resource for QTL mapping can provide enhanced resolution for trait dissection and facilitate the identification of candidate genes underlying resistance mechanisms. Such insights are crucial for developing functional markers that could be implemented in breeding programs and for understanding the biological basis of host-plant resistance to spittlebugs.

The objective of this study was to identify genomic regions associated with host-plant resistance to *Aeneolamia varia* (Fabricius 1787) (Hemiptera: Cercopidae) nymphs in interspecific *Urochloa* hybrids and explore candidate genes that may contribute to understanding the resistance mechanisms.

## Methods

### Plant material and phenotypic data

Four tetraploid interspecific sexual hybrids (*U. ruziziensis* × *U. brizantha* × *U. decumbens*) resistant to *A. varia* were crossed with the common susceptible apomictic tester *U. decumbens* cv. Basilisk (CIAT 606; genome references), comprising 4 F_1_ biparental families with a total of 339 genotypes (Table 1).

**Table 1.** Biparental F_1_ families of *Urochloa* interspecific hybrids.

Cross	Number of individuals of the progeny
Sx14_808 × CIAT 606	70
Sx14_793 × CIAT 606	120
Sx14_123 × CIAT 606	120
Sx14_1042 × CIAT 606	29

The F_1_ progeny was evaluated for resistance to nymphs of *A. varia* (Hemiptera: Cercopidae) in no-choice tests under controlled greenhouse conditions using 2 commercial cultivars as resistant controls (*U.* interspecific cv. Mulato II, and *U. brizantha* cv. Marandú) at the Center for Tropical Agriculture (CIAT), Palmira, Colombia (3°30′12.34″N, 76°21′23.81″W). Following Parsa et al. (2011), the no-choice tests subjected each experimental unit to the same insect infestation rate of 6 *A. varia* eggs close to the hatching date. An experimental unit consists of a single-stemmed plant grown from 1 tiller of the potted plant, with exposed roots to facilitate the insect feeding. The no-choice tests were conducted in 7 independent trials, T1 to T7, each following a Federer augmented-block experimental design with 1 infested and 1 uninfested replicate of the 339 hybrids and the 5 parentals, allocated randomly in each trial (T). The controls were included in each incomplete block. Treatment considered the factor genotype and the factor infestation.

**Fig. 1.**
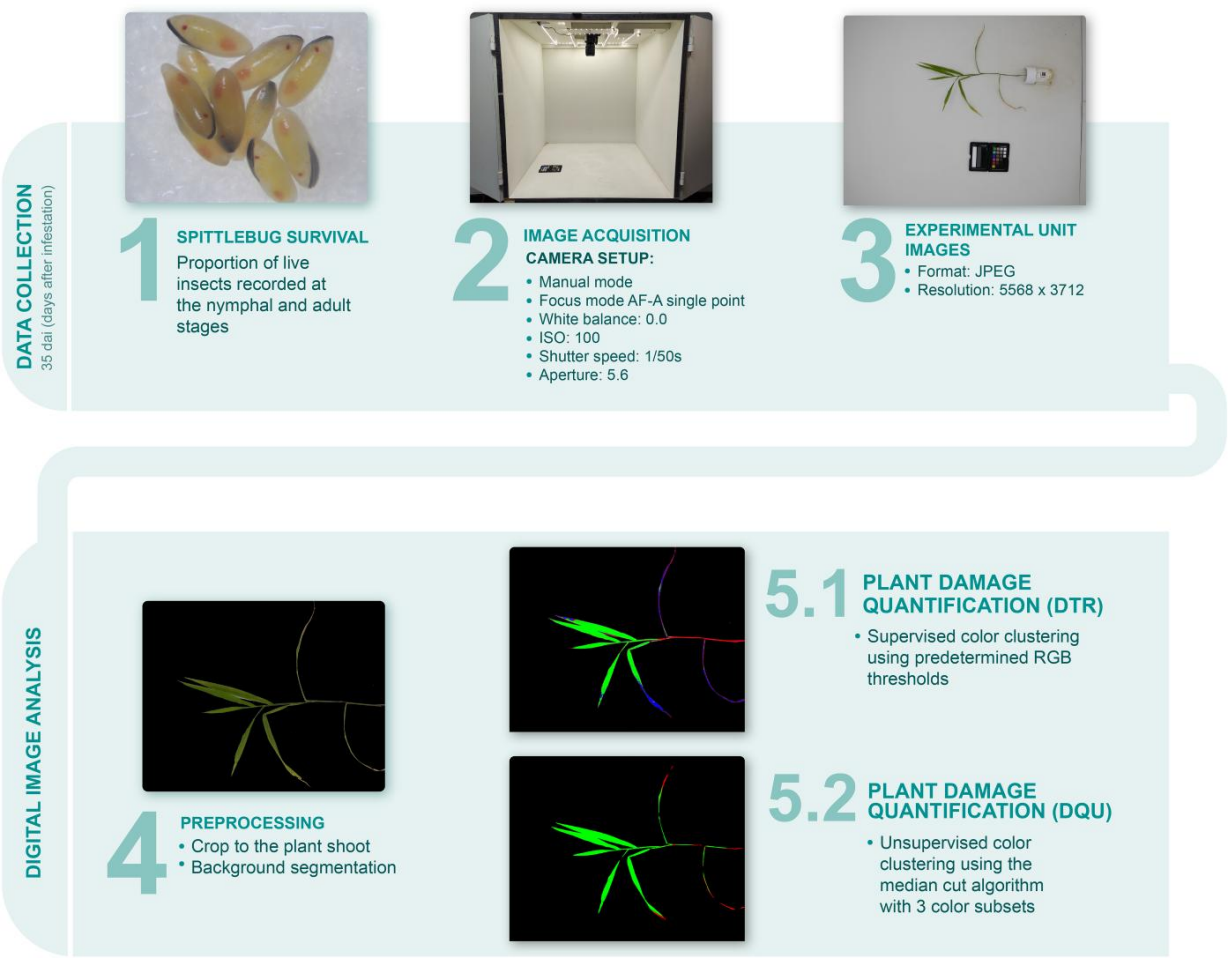
Response variables collected in no-choice trials to *A. varia* nymphal infestation in interspecific *Urochloa* hybrids and digital image analysis workflow to quantify plant damage.

Response variables scored included insect survivorship (%) (NTS) (Cardona et al. 2010) and plant damage (%), for instance, plants with 100% nymph mortality had a 0% of insect survivorship. Both were evaluated at 35 d after infestation (DAI), which aligns with the nymphal stage duration of this spittlebug species (Fig. 1). NTS was evaluated in 5 trials, excluding T1 and T4, and was expressed as the total of living insects in nymphal and adult instars stages at 35 DAI:


$$\mathrm{NTS}=\frac{\;\mathrm{Living}\;\mathrm{insect}\;\mathrm{counts}}{\mathrm{Total}\;\mathrm{infested}\;\mathrm{insects}}\times 100$$

Plant damage percentage was quantified across all trials from digital images acquired in a lightbox under controlled lighting conditions. Images were captured using a Nikon D7500 reflex camera with fixed photographic parameters: AF-A single point focus, white balance 0.0, ISO 100, shutter speed 1/50 s, and aperture f/5.6. Quantification employed 2 separate methodologies. The first, Color Clustering Image Analysis (DQU), utilized an unsupervised color clustering algorithm within ImageJ (Ferreira and Rasband 2012; L. Hernández et al. 2022a). The second, RGB Pixel Counting (DTR), involved using the jpeg package in R (Urbanek 2011; Espitia-Buitrago et al. 2025) to quantify green, chlorotic, and necrotic tissue based on predetermined red, green, and blue (RGB) values. Tissue classification followed these specific criteria: green tissue was defined by R < (0.9 × G) AND B < (0.9 × G) AND (2 × G) > (20/255) × (B + R) (Patrignani and Ochsner 2015); yellow (chlorotic) tissue was classified when (90/255) < R < (241/255) AND (97/255) < G < (216/255) AND (18/255) < B < (165/255); and necrotic tissue was identified when (27/255) < R < (225/255) AND (56/255) < G < (213/255) AND (0/255) < B < (53/255). Finally, plant damage was quantified using the following equation:


$$\mathrm{Damage}=\frac{\;\mathrm{Yellow}\;\mathrm{pixels}\;+\;\mathrm{Necrotic}\;\mathrm{pixels}}{\mathrm{Yellow}\;\mathrm{pixels}+\mathrm{Necrotic}\;\mathrm{pixels}+\mathrm{Green}\;\mathrm{pixels}}$$

### Phenotypic statistical analysis

Phenotypic data from the infested treatment were analyzed using linear mixed models (LMMs) implemented in ASReml-R version 4.3.3 (Butler et al. 2018). Plant damage traits, including total plant damage, were quantified with the unsupervised algorithm (DQU), total plant damage quantified with the supervised algorithm (DTR), and yellow tissue and necrotic tissue quantified with the supervised algorithm (DTR). All metrics were evaluated across all trials. Insect survival (%) (NTS) was analyzed across 5 environments, excluding T1 and T4 where this variable was not recorded due to logistic constraints.

To ensure the data quality, each trail was assessed individually using an LMM with genotype fitted as a random effect to estimate Cullis broad-sense heritability based on the average pairwise prediction error variance between genotypes (Covarrubias-Pazaran 2020). Trials with convergence issues or heritability estimates below 0.2 were excluded from further analysis. The final model incorporated trial (i.e. environment) as a fixed effect and was structured as follows:


$$y=X\beta +Z_{1}u_{1}+Z_{2}u_{2}+Z_{3}u_{3}+Z_{4}u_{4}+\varepsilon$$

where *X* is the design matrix and β is the vector of trial fixed effects; $Z_{i}$ are design matrices for $u_{i}$ vectors: $Z_{1}$ corresponds to genotypic effects $u_{1}$, assumed ∼ N(0, $A\sigma_{g}^{2}$) using a pedigree-derived relationship matrix *A* (build via *A*-inverse using parental records of the hybrids); $Z_{2}$ corresponds to genotype by trial random effects $u_{2}$; $Z_{3}$ corresponds to block random effects $u_{3}$; and $Z_{4}$ corresponds to row × column spatial effects $u_{4}$. Residual errors (ε) were modeled as heterogeneous across trials using dsum(∼units | Trial).

To obtain best linear unbiased predictors (BLUPs) of genotypic performance across trials, genotype was modeled as a random effect. In contrast, best linear unbiased estimators (BLUEs) were calculated by treating genotype as a fixed effect. Cullis broad-sense heritability for each trait was estimated from the BLUP model variance components. Additionally, genotype-specific means were included by the arithmetic averaging of replicated measurements across trials, providing a preliminary measure of phenotypic performance. All estimates, including means, BLUEs, BLUPs, and variance components, were exported for downstream genome-wide association analyses (genome-wide association study [GWAS]). Trait correlations and their significance were assessed using Pearson correlation coefficients calculated from BLUEs, as these represent genotypic values without shrinkage

### Genotypic data and bioinformatic workflow

Leaf samples of the 344 individuals were lyophilized for DNA extraction at Eurofins Genomics. Subsequently, these samples were processed by Floragenex Inc. for single-end RAD-seq using 118 bp reads with the PstI restriction enzyme. To ensure adequate coverage, each F_1_ individual was sequenced twice, while parental samples were sequenced 8 times to achieve higher depth for accurate genotype calling. Raw reads were demultiplexed and quality-trimmed before mapping to the tetraploid *U. decumbens* cv. Basilisk reference genome (GCA_964030465.3) (Ryan et al. 2025) using Bowtie2 algorithm version 2.1 (Langmead and Salzberg 2012). Genotype calling was performed using NGSEP V.3 (Perea et al. 2016) with ploidy set to 2 (diploid mode), as previous studies suggest that mean read depths of at least 61 are required for accurate tetraploid allele dosage estimation in *Urochloa* species, which is higher than the dataset coverage. The resulting VCF file was phased and imputed using Beagle (Browning et al. 2018, 2021) (version 5.5), followed by quality filtering to retain only biallelic SNPs with minor allele frequency (MAF) ≥ 0.01 and call rate ≥ 40%. This filtering pipeline yielded 57,051 high-quality biallelic SNP markers for downstream analyses.

### GWAS and gene annotation

GWAS analysis was conducted in GAPIT version 3 (Wang and Zhang 2021), implementing 2 complementary algorithms: the Fixed and random model Circulating Probability Unification (FarmCPU) (Liu et al. 2016) and the Bayesian-information and Linkage-disequilibrium Iteratively Nested Keyway (BLINK) models (Huang et al. 2019). To account for population structure and relatedness, covariates included the first 3 principal components and a kinship matrix calculated using the VanRaden method (VanRaden 2008). Phenotypic data consisted of estimates per genotype derived from the LMM analyses for plant damage using both the DQU and DTR methodologies, as well as insect survival (%) (NTS). Genotypic data consisted of a quality-filtered VCF containing 57,051 biallelic SNPs.

Significant marker–trait associations (MTAs) were identified using a −log_10_(*P*-value) threshold of 6 and validated through false discovery rate correction using the Benjamini–Hochberg procedure, as implemented in GAPIT (Wang and Zhang 2021). Quantitative trait loci (QTL) regions were then defined by applying 3 window sizes (±2 kb, ±5 kb, and ±10 kb) around each significant MTA, based on the estimated linkage disequilibrium (LD) decay distance (*r*^2^ = 0.2) for each biparental populations. LD decay analysis was performed using PopLDdecay v1.4.4 (Zhang et al. 2019) with a MAF threshold of 0.01. Pairwise LD values (*r*^2^) were calculated between all SNP pairs and averaged across fixed genomic distance bins of 1 kb to generate decay curves per chromosome. The LD decay distance was estimated as the point where the fitted decay curve intersected *r*^2^ = 0.2, for each F1 family and for the general population. Candidate genes located within the defined windows were extracted from the *U. decumbens* cv. Basilisk reference genome (GCA_964030465.3) (Ryan et al. 2025) and functionally annotated using BLASTp searches against the UniProt database (The UniProt Consortium 2025). Searches were conducted with default parameters, except for an *E*-value threshold of 0.01. Results were filtered to retain only hits with ≥85% identity, optimal alignment length coverage ≥ 70%, and high-confidence functional annotations (UniProt Protein Existence evidence levels 1 or 2: “Evidence at protein level” or “Evidence at transcript level”).

### Use of large language models

Portions of the manuscript text and R code for data visualization were refined with assistance from ChatGPT (OpenAI GPT-4, version released July 2023, accessed via https://chat.openai.com/). The model was queried using natural language prompts to improve the clarity of scientific writing and to polish R code used for generating graphs and figures. All model outputs were critically reviewed, edited, and validated by the authors to ensure scientific accuracy, proper formatting, and originality.

## Results

### Phenotypic analysis

**Fig. 2.**
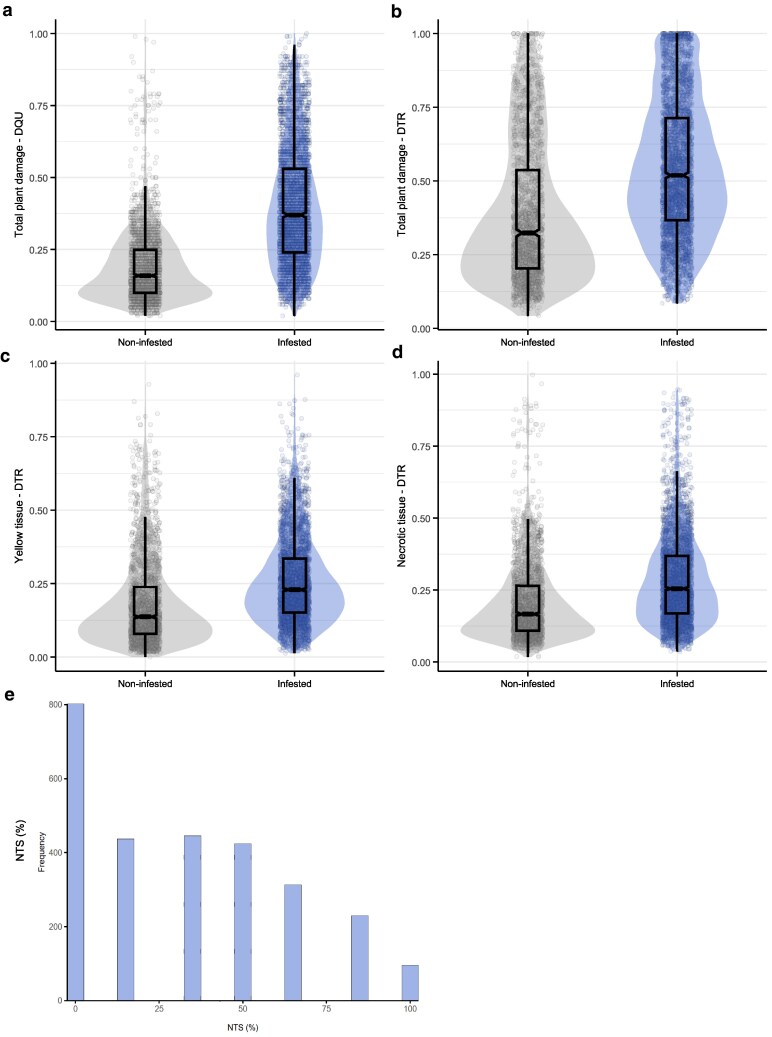
Violin plots displaying the distribution of raw plant damage variables and histogram displaying insect survival across the 7 trials.

A high degree of phenotypic variation in plant damage and insect survival was observed among the *Urochloa* interspecific population in response to *A. varia* nymph infestation (Supplementary Table 1). Infested plants showed higher levels of damage compared to non-infested plants across all the image-derived variables, regardless of the quantification method used, either DQU (unsupervised clustering with color quantization) or DTR (supervised clustering with RGB thresholds) (Fig. 2, a to d; Supplementary Fig. 1). In contrast, insect survival (NTS), calculated as the proportion of live insects 35 DAI, was evaluated as a standalone biological variable independent of the digital models (Fig. 2e) and showed discrete distribution due to its derivation from fixed insect counts.

**Fig. 3.**
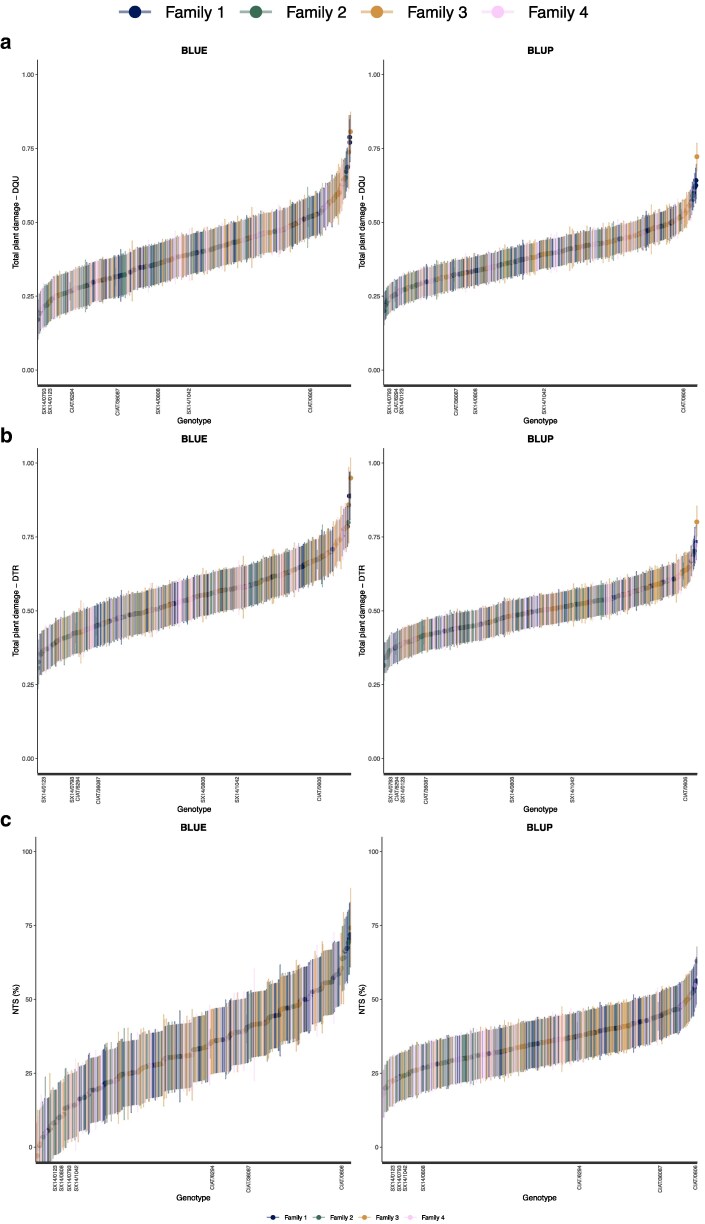
BLUEs and BLUPs of total plant damage calculated with a) unsupervised median cut algorithm for color quantization using 3 color subgroups (DQU); b) supervised RGB threshold setting (DTR); and c) insect survival (NTS %).

**Fig. 4.**
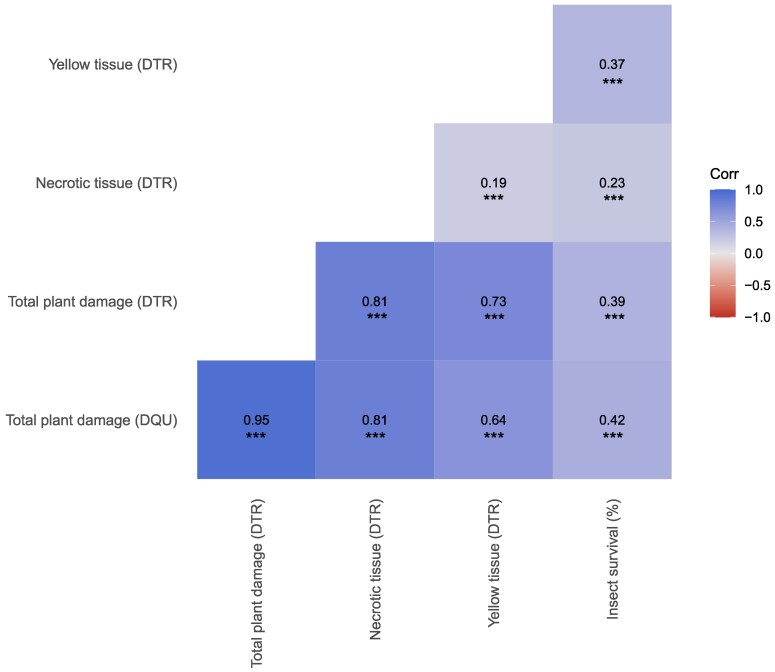
Pearson correlation matrix among BLUEs of plant damage traits and insect survival (%). Significance levels indicated by asterisks (***P < 0.001).

Trial-level analyses of broad-sense heritability revealed that trial T2 consistently showed low estimates (H^2^ < 0.2) across all evaluated traits and was therefore excluded from final statistical analyses to ensure model stability and data quality (Supplementary Table 2). Notably, damage estimates were consistently higher with the DTR method compared to DQU; however, genotype classification remained consistent between both image analysis techniques (Fig. 3). A strong and highly significant correlation was observed between total plant damage estimates derived from DQU and DTR (*r* = 0.95), as well as with necrotic tissue (*r* = 0.81). Correlations between total damage and yellow tissue were slightly lower, ranging from *r* = 0.64 for the DQU method to *r* = 0.73 for DTR. In contrast, insect survival NTS exhibited low to moderate correlations with all damage traits (Fig. 4).

Broad-sense heritability estimates of plant damage traits ranged from moderate to high, with values from 0.49 for necrotic area (DTR) to 0.66 for total plant damage (DQU) (Table 2). NTS showed the lowest heritability estimate (0.42), reflecting the effect of its discrete, bounded nature and the high frequency of 0 survival, i.e. 0% of insect survivorship, observed at 35 DAI.

**Table 2.** Cullis broad-sense heritability estimates for phenotypic traits in the interspecific *Urochloa* population.

Trait	Cullis broad-sense heritability
Total plant damage (DQU)	0.660
Total plant damage (DTR)	0.609
Yellow tissue (DTR)	0.558
Necrotic tissue (DTR)	0.490
Insect survival (%) (NTS)	0.428

### GWAS analysis

**Fig. 5.**
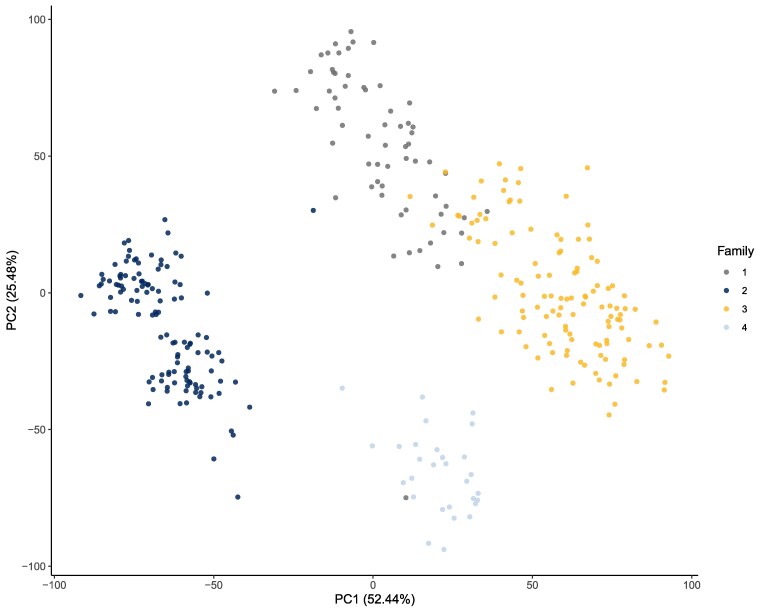
Principal component analysis plot from the first 2 principal components.

Principal component analysis revealed a clear population structure (Supplementary Table 3), with the first 2 principal components explaining 77.92% of the total genetic variance (Fig. 5). Individuals clustered according to their biparental family, supporting the presence of stratification consistent with the controlled cross design. To control this structure in the GWAS models, the first 3 principal components, which cumulatively explained 77.92% of the genetic variance, were included as covariates in FarmCPU and BLINK models.

**Fig. 6.**
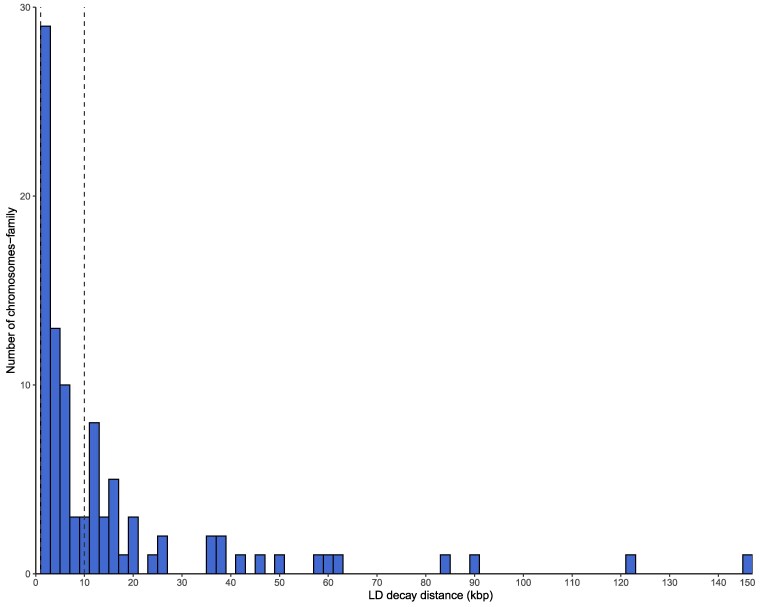
Distribution of LD decay distances (*r*^2^ = 0.2) across chromosomes in the F_1_ mapping population. Vertical dashed lines indicate the 1 and 10 kb reference thresholds used to guide QTL window size selection.

LD analysis for the entire F_1_ population revealed that LD decayed to *r*^2^ = 0.2 at approximately 2,000 bp. However, substantial variation in LD decay patterns was observed among the 4 biparental families. While families 1 to 3 showed similar LD decay distances of ∼1,000 bp, family 4 exhibited extended LD persistence up to ∼3,000 bp. Across chromosomes, LD decay distances (*r*^2^ < 0.2) ranged from 1 kb to ∼88 kb, with most chromosomes exhibiting decay within 2 to 10 kb (Fig. 6). Given the heterogeneous LD patterns among families, we opted to use 3 window sizes (±2 kb, ±5 kb, and ±10 kb) around each significant MTA for candidate gene identification.

**Fig. 7.**
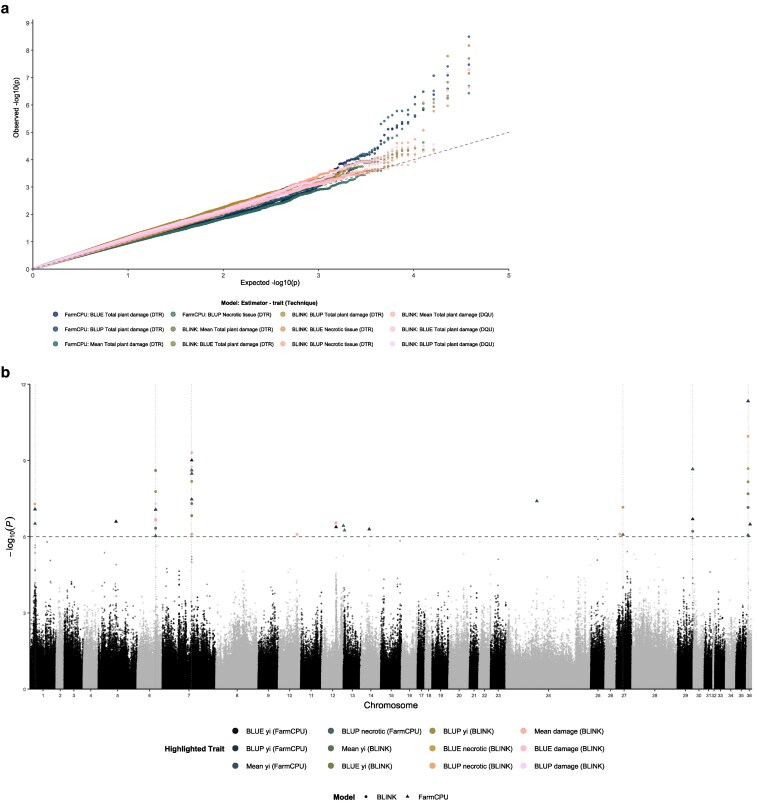
Manhattan plot and Q-Q plot of SNP associations with plant damage traits using BLINK and FarmCPU models. The *x* axis represents the genomic position of the markers, and the *y* axis shows the –log_10_(*P*) values for each SNP. The dashed horizontal line indicates the genome-wide significance threshold (−log_10_(*P*-value) = 6). Dotted vertical lines mark chromosomes harboring clusters of significant associations. Different colors and symbols represent trait-model combinations: DTR (plant damage rating) and DQU (damage qualification units) assessed using both BLINK and FarmCPU algorithms.

Genome-wide association analysis did not identify significant associations with insect survival (NS) but revealed 46 significant MTAs for plant damage phenotypes (necrotic tissue or total plant damage), all exceeding the threshold of −log_10_(*P*-value) > 6, using both FarmCPU and BLINK models in GAPIT (Fig. 7; Supplementary Table 4). Q-Q plots demonstrated good model performance with minimal deviation from the expected null distribution and controlled genomic inflation (λ ≈ 1.0), indicating that population structure and kinship were adequately controlled by the inclusion of principal components and kinship matrix as covariates (Fig. 7).

Eighteen consistent and robust QTLs were detected for total plant damage assessed through the DQU methodology, with additional significant associations identified for necrotic tissue damage using the DTR approach (Table 3). Among them, 6 high-confidence QTLs were located on chromosomes 1, 6, 7, 27, 29, and 36 and consistently detected across both statistical models, multiple phenotypic estimators, and both plant damage quantification methodologies, with overlapping significant MTAs (Table 3). These robust QTLs likely represent genetic variants with stable and reproducible effects on response to plant damage to *A. varia* nymph feeding damage, suggesting multiple independent genetic mechanisms underlying this complex resistance trait. Among these, a locus on chromosome 7 (position 28,519,388) exhibited the largest negative effect size (β = –0.0532) and explained the highest proportion of phenotypic variance (PVE = 21.5%) for spittlebug resistance. However, this locus was associated with a rare favorable allele (MAF = 0.0738), suggesting limited frequency in the studied population. In contrast, a SNP on chromosome 1 (position 11,524,270) displayed a moderate effect (β = 0.0235) coupled with a high allele frequency (MAF = 0.483), making it particularly attractive to try for marker-assisted selection in the breeding population. Additional recurrent loci on chromosomes 27, 29, and 36 demonstrated moderate to small effect sizes (β = 0.00903 to 0.0272) with intermediate allele frequencies (MAF = 0.254 to 0.388), suggesting these variants could contribute additively to spittlebug resistance to plant damage.

**Table 3.** Identified QTL for plant damage traits in the interspecific *Urochloa* population.

Name	Chromosome	Start	End	Trait
qNT1	1	11334775	11354775	Necrotic tissue (DTR)
qTPD1	1	11514270	11534270	Total plant damage (DQU)
qTPD5	5	63376517	63396517	Total plant damage (DQU)
qTPD6	6	64514508	64534508	Total plant damage (DQU); total plant damage (DTR); necrotic tissue (DTR)
qTPD7	7	28509388	28529388	Total plant damage (DQU); total plant damage (DTR); necrotic tissue (DTR)
qTPD10	10	58791952	58811952	Total plant damage (DTR)
qTPD12-1	12	54702901	54722901	Total plant damage (DTR)
qTPD12-2	12	56104516	56124516	Total plant damage (DTR)
qTPD12-3	12	56374467	56394467	Total plant damage (DQU)
qNT12	12	82648095	82668095	Necrotic tissue (DTR)
qNT13	13	3083711	3103711	Necrotic tissue (DTR)
qTPD14	14	23526073	23546073	Total plant damage (DQU)
qTPD24	24	52838791	52858791	Total plant damage (DQU)
qNT27-1	27	13940992	13960992	Necrotic tissue (DTR)
qNT27-2	27	31335868	31355868	Necrotic tissue (DTR)
qTPD29	29	57321694	57341694	Total plant damage (DQU)
qTPD36-1	36	15980330	16000330	Total plant damage (DQU); total plant damage (DTR); necrotic tissue (DTR)
qTPD36-2	36	37621131	37641131	Total plant damage (DQU)

The functional relevance of the identified QTL was assessed through positional and protein-based annotation. A total of 34 candidate genes were identified within the ±2 kb, ±5 kb, and ±10 kb windows surrounding significant MTAs based on the *U. decumbens* genome annotation (Supplementary Table 5). Initial BLASTp searches against the UniProt database detected 2,101 proteins matches, but after applying stringent filtering criteria (≥85% sequence identity and Protein Existence levels 1 or 2 in UniProt), 30 high-confidence candidate proteins were retained as putative functional genes. These candidates were confirmed to be located within the specific genomic intervals of the identified QTLs or in high LD with the lead SNPs on chromosomes 1, 5, 10, 12, 13, 14, and 27 (Table 4). Among the 6 high-confidence MTAs identified across both GWAS models, only the SNP on chromosome 1 (position 11,524,270) was within a high-confidence annotated protein, while the remaining 5 robust MTAs on chromosomes 6,7,27,29 and 36 were located in genomic regions lacking well-annotated protein-coding sequences.

**Table 4.** Significant MTAs for spittlebug plant damage response identified through GWAS analysis.

QTL	*Urochloa* gene name UniProt	Start	End	Protein	Main function	Related function with resistance	References
qNT1	LOCUS_993	11332693	11334978	Protein ELC-like	Signaling and regulation	Found in the ESCRT complex: cytokinesis, regulation of vesicular trafficking process, cellular autophagy, nuclear envelope reformation, membrane repair, ABA regulation	Spitzer et al. (2006); Yu et al. (2020)
qNT1	LOCUS_994	11335110	11338139	Uncharacterized protein	…	…	…
qTPD1	LOCUS_997	11345300	11348404	Rac-like GTP-binding protein 5	Signaling and regulation	Key regulator of immunity responses, cell death, and production of ROS	Kawano et al. (2014); Kawasaki et al. (1999)
qTPD1	LOCUS_997	11345300	11348404	Rac-like GTP-binding protein 5			
qTPD5	LOCUS_1012	11512420	11514969	Glutathione peroxidase	Antioxidant and metabolic enzyme	Eliminate ROS to prevent cellular damage, protect against cell death, ABA signaling pathway, senescence delaying, and reinforcement of physical barriers	Zechmann (2020)
qTPD10	LOCUS_1012	11512420	11514969				
qTPD12-1	LOCUS_1012	11512420	11514969				
qTPD12-2	LOCUS_1013	11530189	11532991	Phosphoribosyl anthranilate transferase	Antioxidant and metabolic enzyme	Related to shikimate pathway: production of aromatic amino acids such as tryptophane, production of secondary metabolites involved in plant defense	Lambrecht and Downs (2013); Scully et al. (2023)
qTPD12-3	LOCUS_100248	63384000	63386212	RING/U-box superfamily protein	Signaling and regulation	Found in UPS: targeting proteins for ubiquitination, regulation of cell cycle and senescence, regulation of ABA-dependent stomatal responses	Karthik et al. (2025); González-Lamothe et al. (2006)
qNT12	LOCUS_6034	58792240	58794245	Cell wall hydroxyproline-rich glycoprotein	Cell wall structure and barrier	Contribute to the cell wall strengthening and maintain the structure, inhibit the pathogenic cell wall degrading enzymes	Deepak et al. (2010); Yuan-Yuan et al. (2018)
qNT13	LOCUS_12866	54706751	54714980	Oxidoreductase, 2OG-Fe oxygenase family protein	Antioxidant and metabolic enzyme	Involved in oxidation reactions, biosynthesis of phytohormones, jasmonic acid signaling pathway	Wang et al. (2021); Zheng et al. (2022)
qTPD14	LOCUS_12866	54706751	54714980	Fe2OG dioxygenase domain-containing protein			
qNT27-1	LOCUS_12969	56112494	56115313	Transmembrane protein	…	…	…
qNT1	LOCUS_12970	56120961	56124563	Anther-specific proline-rich protein APG	Other	Cell wall modification and pollen development. Found in pathogen attack	Roberts et al. (1993); Lee and Cho (2003)
qNT1	LOCUS_12990	56390023	56394977	F-box protein FBL2	Signaling and regulation	Found in UPS: targeting proteins for ubiquitination, regulation of cell cycle and senescence, phytohormones pathway including jasmonic acid	Karthik et al. (2025); Abd-Hamid et al. (2020)
qTPD1	LOCUS_15310	82654510	82657464	Very-long-chain aldehyde decarbonylase	Cell wall structure and barrier	Biosynthesis of cuticular waxes	Pascal et al. (2019); Aragón et al. (2021)
qTPD1	LOCUS_15310	82654510	82657464	Aldehyde oxygenase (deformylating)			
qTPD5	LOCUS_15765	3097586	3101544	Type I inositol-1,4,5-trisphosphate 5-phosphatase	Signaling and regulation	Modulate intracellular calcium levels for signaling defense mechanisms such as movement of stomatal guard cells; involved on systemic acquired resistance	Huang et al. (2019); Perera et al. (2008)
qTPD10	LOCUS_22598	23536761	23540288	Plant intracellular Ras-group-related LRR protein 4	Signaling and regulation	Signal transduction and pathogen recognition, cell death regulation	Forsthoefel et al. (2005)
qTPD12-1	LOCUS_68004	13942135	13949209	Mitosis protein dim1	Other	Mitotic progression and chromosome segregation, pre-mRNA splicing	Simeoni and Divita (2007); Berry and Gould (1997)
qTPD12-2	LOCUS_68004	13942135	13949209	DIM1			

The table includes GAPIT model parameters, genomic positions, and functional annotated candidate genes with UniProt functional information.

## Discussion

For over 2 decades, the interspecific *Urochloa* breeding program at the International Center for Tropical Agriculture (CIAT) has prioritized the evaluation of resistance as core strategies to breed for spittlebug resistance, achieving substantial genetic gains (Worthington and Miles 2015; Hernández et al. 2022b). Dissecting the genetic architecture underlying these resistance mechanisms in each breeding cycle is, therefore, essential to sustain and enhance these gains, considering the genetic diversity and adaptability observed in spittlebug populations (Hernández et al. 2021). Our GWAS provide molecular evidence supporting the importance of evaluating both resistance categories independently. The weak phenotypic correlation between insect survival (NTS) and plant damage traits, together with the absence of significant associations for insect survival (antibiosis), contrasted with the identification of 18 significant MTAs for plant damage traits, suggests these 2 mechanisms are controlled by distinct and independent genetic networks. These findings align with the principle that plant resistance to herbivorous insects is often polygenic and continuously shaped by ongoing coevolution with genetically diverse pest populations (Demirjian et al. 2023). Thus, integrating resistance mechanisms can contribute to long-term, durable, and broad-spectrum resistance in *Urochloa* breeding populations (Priyadarshan 2019).

The identification of resistance to plant damage-specific QTL provides a foundation for marker-assisted selection approaches that can complement phenotypic evaluation methods that quantify the level of plant damage caused by spittlebugs nymphs through image analysis, accelerating the development of tolerant *Urochloa* cultivars. Insect survival (NTS) exhibited moderate heritability (H^2^ = 0.42), yet no significant SNP associations were identified in our GWAS, highlighting the challenges of detecting genetic signals for traits derived from discrete measurements. In this study, the limited genetic signal observed for NTS likely reflects the statistical complications arising from its derivation from insect counts, which were characterized by non-normal distributions and bounded outcomes constrained to discrete intervals (e.g. 0%, 16.7%, 33.3%). As described by Reid and Acker (2022), such distribution reduces statistical power and limits the capacity to fully capture the underlying genetic variance contributing to the trait, consequently, yielding lower estimates of broad-sense heritability. Similar challenges have been reported by Ferreira et al. (2019), who identified 3 QTL explaining less than 6.5% of phenotypic variance in *Notozulia entreriana* nymph survival (H^2^ = 0.37) in a *U. decumbens* intraspecific population with an allele dosage-based linkage mapping approach. The authors attributed these modest effects to the limited resistance of both parentals, insufficient sequencing depth, and methodological constraints intrinsic to reduced-representation genotyping; these latter factors likewise account for the lack of NTS-associated SNPs in the present study.

The use of single-end reduced-representation sequencing (RAD-seq), while cost-effective for large populations, constrains marker density and genotyping resolution, particularly given the segmental allotetraploid nature of *U. decumbens* and the uncharacterized meiotic behavior of the hybrids (Worthington et al. 2016; Njuguna et al. 2023; Ryan et al. 2025). For this reason, standard diploid genotype calling approaches may not adequately capture the true allelic relationships and segregation patterns underlying antibiosis traits, further complicating the detection of genetic associations.

On the other hand, plant damage traits exhibited substantially higher heritability estimates (up to H^2^ = 0.66 for total damage assessed via DQU), supporting the accuracy of quantification from image-based phenotyping for capturing phenotypic variability within the hybrid *Urochloa* population and delivering a continuous variable. The integration of 2 digital analysis methods, DQU (unsupervised clustering) and DTR (supervised clustering), allowed the quantification of 4,816 images with high precision and speed, without requiring expert evaluation, processing 100 segmented JPEG images in just 4 min and 34 s (DTR) and 3 min and 47 s (DQU) on a single processor core. Additionally, the combined use of DTR and DQU methodologies for measuring total plant damage and necrotic areas strengthened the identification of MTAs by providing complementary phenotypic perspectives on resistance to plant damage mechanisms. This dual methodology approach enhanced the reliability of our results, as evidenced by the consistency of significant SNP associations detected across both statistical models (FarmCPU and BLINK) and phenotypic methods. This efficiency and scalability are particularly advantageous for GWAS, where precise and continuous phenotypes enhance statistical power to detect MTAs by improving phenotypic resolution across environments and experimental trails in our case, outperforming traditional categorical visual scoring scales in the dissection of complex traits such as resistance to plant damage (Araus et al. 2018; McDonald et al. 2022; Xiao et al. 2022).

Overall, and in accordance with previous findings in *Urochloa* for spittlebug resistance (Ferreira et al. 2019) and forage yield-related traits (Vidotti et al. 2019), the loci associated with plant damage explained moderate proportions of phenotypic variance (PVE < 22%). Among the robust set of 6 markers, 3 SNPs on chromosomes 6, 7, and 36 stood out due to their recurrence across multiple models, estimators, and plant damage traits. The SNP on chromosome 7 (position 28,519,388) exhibited the highest phenotypic variance explained (PVE = 21.5%), the largest effect size (β = −0.0532), while being consistently detected across all trait quantifications (DQU, DTR, and necrotic tissue). However, its low MAF (MAF = 0.07) indicates that the favorable allele is rare within the current breeding population. This potentially limits its applicability by itself in marker-assisted selection, particularly for populations from other breeding cycles where the allele may be rare or absent. In contrast, SNPs on chromosome 6 (position 64,524,508) and chromosome 36 (position 15,990,330) showed moderate phenotypic variance explained and effect (PVE ≈ 5.4%) but higher allele frequencies (MAF = 0.34 and 0.25, respectively), making them more immediately accessible for breeding applications. These moderate effect loci, while individually contributing smaller phenotypic improvements, offer greater potential for practical implementation due to their higher frequency of favorable alleles.

In cassava, consistent GWAS peaks, detected across multiple statistical approaches for the same whitefly (*Aleurotrachelus socialis*) resistance trait, lead to the development of 3 high-association SNP markers, with one successfully validated for operational marker-assisted selection in the CIAT's breeding program (Bohorquez-Chaux et al. 2025).

The 18 MTAs identified in this study represent the first set of markers available for spittlebug resistance to plant damage in *Urochloa* grasses, providing a foundation for implementing genomic-assisted breeding strategies. Given the moderate effect sizes and varying allele frequencies of these markers, a targeted approach focusing on the most robust associations (particularly those on chromosomes 1, 6, 7, 27, 29 and 36) would be most practical for marker development and validation, e.g. using competitive amplification such as KASP.

Our findings support the complex and polygenic nature of spittlebug resistance in *Urochloa*. As described by Peterson et al. (2017), insect tolerance is typically governed by multiple loci, each contributing a modest, individual effect through poorly characterized mechanisms, that are often influenced by the intensity of herbivore pressure and involve a limited number of transcripts functionally linked to resistance at the molecular level. In hemipterans’ resistance systems, the mechanisms related to both constitutive and induced resistance have been documented, including compensation of photosynthetic activity, upregulation of peroxidases and oxidative enzymes, and elevation of phytohormone levels (Koch et al. 2016).

Candidate proteins functionally related to these established resistance mechanisms were identified in our QTL, providing valuable insights into their putative roles in the response to plant damage to *A. varia*'s feeding damage in *Urochloa.* These candidates represent a diverse network of protein families spanning multiple functional categories: signaling and regulatory proteins involved in stress perception and response coordination, antioxidant and metabolic enzymes responsible for cellular protection and metabolic adjustment, and structural proteins contributing to cell wall reinforcement and physical barriers (Table 4). This functional diversity reflects the multifaceted nature of plant responses to spittlebug herbivory and suggests that responses to plant damage mechanisms in *Urochloa* involve coordinated activation of multiple defense pathways rather than reliance on single resistance genes.

Among the signaling and regulatory proteins, several candidates are likely involved in modulating immune responses, hormone signaling pathways, and stress-induced cell death regulation. More specifically, these proteins are implicated in modulating defense signaling cascades and programmed cell death, as well as coordinating hormone responses, particularly those involving ABA and JA—key phytohormones in the response to herbivory (Kawasaki et al. 1999; Forsthoefel et al. 2005; González-Lamothe et al. 2006; Spitzer et al. 2006; Perera et al. 2008; Kawano et al. 2014; Lannoo and Van Damme 2014; Abd-Hamid et al. 2020; Yu et al. 2020; Karthik et al. 2025). These proteins often act as intracellular mediators that perceive or transduce signals following herbivory, contributing to plant resistance responses in different stages of pest attack. The identification of hormone signaling components is particularly relevant, as JA-mediated responses are central to induced defenses against chewing insects, while ABA pathways regulate stress tolerance and resource allocation during herbivore pressure (Ballaré 2011; Wang and Wu 2013).

The antioxidant and metabolic enzymes group includes candidates with established roles in mitigating oxidative stress, maintaining cellular homeostasis, and supporting the biosynthesis of secondary metabolites. These proteins contribute to detoxifying reactive oxygen species (ROS) generated during herbivore attack, synthesizing defensive phytohormones, and modulating calcium signaling and systemic acquired resistance pathways, which are frequently activated during insect herbivory (Lambrecht and Downs 2013; Zechmann 2020; Wang et al. 2021; Zheng et al. 2022; Scully et al. 2023; do Carmo Santos et al. 2025). The presence of oxidative stress response proteins is particularly significant, as spittlebug feeding can disrupt normal cellular processes and generate harmful ROS that must be neutralized to prevent cellular damage and maintain photosynthetic efficiency (Pacheco-Coeto et al. 2019).

In parallel, structural and barrier-related proteins were identified that are likely involved in reinforcing the plant physical defenses. These include enzymes and proteins associated with cell wall fortification, cuticle development, and extracellular matrix stability. By enhancing cell wall strengthening and promoting deposition of protective surface layers, these components serve as a primary line of defense, potentially limiting spittlebug penetration and reducing feeding success (Deepak et al. 2010; Yuan-Yuan et al. 2018; Pascal et al. 2019; Aragón et al. 2021). The identification of cell wall modification enzymes is particularly relevant for spittlebug resistance to plant damage, as these insects must penetrate plant tissues to access vascular elements for feeding (Begnami et al. 2025).

Some protein families identified in our study overlap with those reported by Ferreira et al. (2019) including LRR-containing proteins and F-box domain proteins, both involved in defense signaling and protein degradation pathways. These shared findings reinforce their potential role in resistance mechanisms against spittlebugs. However, they also identified WRKY transcription factors, NB-ARC proteins, and pathogenesis-related proteins that were not detected in our analysis, likely reflecting differences in mapping approaches, population genetics, target pest species, or the specific resistance mechanisms being evaluated. Conversely, our study identified additional candidates related to oxidative stress mitigation, calcium signaling, and cuticle biosynthesis, suggesting complementary physiological mechanisms specifically involved in *A. varia* nymph resistance to plant damage. A few candidate proteins did not show a direct relationship to established plant defense mechanisms. These included proteins involved in general cellular functions such as membrane transport, pollen development, and cell division. For example, one candidate associated with pollen-specific expression and cell wall modification has previously been reported in unrelated plant–pathogen interactions, suggesting a possible role in defense-related signaling (Roberts et al. 1993; Lee and Cho 2003). Another candidate involved in mitotic progression and pre-mRNA splicing may reflect either baseline cellular maintenance requirements during stress rather than a targeted response to herbivory (Berry and Gould 1997; Simeoni and Divita 2007). While their primary functions are not defense-related, these proteins may participate to indirect roles in broader physiological adjustments that support plant survival under herbivore pressure, or they may simply be in LD with unmapped causal loci.

Additionally, while not all significant SNPs mapped directly to annotated genes, this outcome is expected given the use of reduced-representation sequencing (RAD-seq), which captures only a fraction of the genome. In GWAS, especially those based on RAD-seq, significant SNPs often represent markers in LD with actual causal variants rather than being causative themselves (Pudjihartono et al. 2022). This is especially relevant in complex polyploid genomes like *Urochloa*, where regulatory elements, non-coding RNAs, or structural variants in intergenic regions may contribute to trait variation. Therefore, these SNPs should be interpreted as valuable markers linked to functional loci that can guide fine-mapping efforts and serve as informative proxies for underlying resistance mechanisms in breeding applications.

The candidate genes identified in this study provide a foundation for several important future research directions that could enhance our understanding of spittlebug resistance mechanisms and their practical application in the forages breeding program. From a breeding perspective, the functional diversity identified across signaling, metabolic, and structural protein categories suggests that pyramiding alleles from different functional pathways could enhance resistance durability and breadth, potentially providing more robust protection against evolving spittlebug populations and varying environmental pressures (Pilet-Nayel et al. 2017). This multi-pathway approach has shown success in other crop-pest systems, where combining different resistance mechanisms reduces the likelihood of pest adaptation (Poland et al. 2009).

For functional validation priorities, candidates involved in hormone signaling (particularly JA and ABA pathways) and oxidative stress responses should be prioritized, as these represent well-characterized defense mechanisms. Furthermore, the identification of SNPs in intergenic regions highlights the potential importance of regulatory variants and non-coding RNAs in controlling plant damage responses, suggesting that fine-mapping efforts using higher-density markers or whole-genome sequencing could resolve the molecular basis of these associations and identify cis-regulatory elements that modulate candidate gene expression.

The MTAs identified in this study constitute a valuable contribution for inclusion in the strategic design of a mid-density SNP panel currently under development for genomic selection in the forages breeding program. Rather than relying solely on genome-wide marker distribution, incorporating the 18 spittlebug response to plant damage-associated SNPs, particularly the 6 high-confidence markers on chromosomes 1, 6, 7, 27, 29, and 36, into the panel design would ensure that key resistance loci are directly captured rather than relying on LD with flanking markers. Additionally, incorporating SNPs from the broader QTL regions (±10 kb windows) around each MTA would provide redundancy and capture potential regulatory variants that may contribute to the observed associations. This strategy of combining trait-specific markers with genome-wide coverage has proven effective in other crop genomic selection programs, improving prediction accuracy for target traits while maintaining broad genetic coverage (Anilkumar et al. 2023; Verma et al. 2024).

The integration of these spittlebug related markers into the mid-density panel would enable simultaneous selection for multiple agronomic traits while ensuring that resistance mechanisms are not inadvertently selected against during genomic selection for yield or quality traits. Furthermore, including these validated markers would provide benchmarks for calibrating genomic prediction models and enable the development of weighted selection indices that appropriately balance spittlebug resistance with other breeding objectives in the *Urochloa* improvement program (Strandén and Jenko 2024).

Ultimately, integrating a high-throughput phenotyping methodology and molecular marker-based strategies provide a robust framework for improving *Urochloa* resistance to *A. varia*, supporting the development of improved cultivars for tropical and subtropical livestock production systems.

## Supplementary Material

jkag101_Supplementary_Data

## Data Availability

The sequencing (RAD-Seq) dataset has been deposited and is available under accession number PRJEB109285 (https://www.ebi.ac.uk/ena/browser/view/PRJEB109285). The digital images used for plant damage quantification are available in the Harvard Dataverse repository at the following identifier: https://dataverse.harvard.edu/dataset.xhtml?persistentId=doi:10.7910/DVN/EGUVHA. Supplemental material available at G3 online.

## References

[jkag101-B1] Abd-Hamid NA, Ahmad-Fauzi MI, Zainal Z, Ismail I. 2020. Diverse and dynamic roles of F-box proteins in plant biology. Planta. 251:68. 10.1007/s00425-020-03356-8. 32072251

[jkag101-B2] Alvarenga R, Auad AM, Moraes JC, da Silva SEB, Rodrigues BS. 2019. Tolerance to nymphs and adults of *Mahanarva spectabilis* (Hemiptera: Cercopidae) by forage plants in fertilized soils. Pest Manag Sci. 75:2242–2250. 10.1002/ps.5361. 30701648

[jkag101-B3] Anilkumar C et al 2023. Gene based markers improve precision of genome-wide association studies and accuracy of genomic predictions in rice breeding. Heredity (Edinb). 130:335–345. 10.1038/s41437-023-00599-5. 36792661 PMC10163052

[jkag101-B4] Aragón W, Formey D, Aviles-Baltazar NY, Torres M, Serrano M. 2021. *Arabidopsis thaliana* cuticle composition contributes to differential defense response to *Botrytis cinerea*. Front Plant Sci. 12:738949. 10.3389/fpls.2021.738949. 34804086 PMC8603936

[jkag101-B5] Araus JL, Kefauver SC, Zaman-Allah M, Olsen MS, Cairns JE. 2018. Translating high-throughput phenotyping into genetic gain. Trends Plant Sci. 23:451–466. 10.1016/j.tplants.2018.02.001. 29555431 PMC5931794

[jkag101-B6] Ballaré CL . 2011. Jasmonate-induced defenses: a tale of intelligence, collaborators and rascals. Trends Plant Sci. 16:249–257. 10.1016/j.tplants.2010.12.001. 21216178

[jkag101-B7] Barros RDA et al 2021. Differential defense responses of tropical grasses to *Mahanarva spectab*ilis (Hemiptera: Cercopidae) infestation. An Acad Bras Cienc. 93:. 10.1590/0001-3765202120191456. 34378641

[jkag101-B8] Begnami IdS et al 2025. Elucidating molecular responses to spittlebug attack in *Paspalum regnellii*. Plant Mol Biol Rep. 43:307–323. 10.1007/s11105-024-01487-w.

[jkag101-B9] Berry LD, Gould KL. 1997. Fission yeast *dim1^+^* encodes a functionally conserved polypeptide essential for mitosis. J Cell Biol. 137:1337–1354. 10.1083/jcb.137.6.1337. 9182666 PMC2132542

[jkag101-B10] Bohorquez-Chaux A et al 2025. Genetic mapping and validation of QTL for whitefly resistance in cassava (*Manihot esculenta* Crantz). Theor Appl Genet. 138:160. 10.1007/s00122-025-04949-1. 40555841 PMC12187898

[jkag101-B11] Browning BL, Tian X, Zhou Y, Browning SR. 2021. Fast two-stage phasing of large-scale sequence data. Am J Hum Genet. 108:1880–1890. 10.1016/j.ajhg.2021.08.005. 34478634 PMC8551421

[jkag101-B12] Browning BL, Zhou Y, Browning SR. 2018. A one-penny imputed genome from next-generation reference panels. Am J Hum Genet. 103:338–348. 10.1016/j.ajhg.2018.07.015. 30100085 PMC6128308

[jkag101-B13] Butler D, Cullis BR, Gilmour AR, Gogel BJ, Thompson R. 2018. ASReml-R reference manual version 4 (4.3.3). VSN International Ltd.

[jkag101-B14] Cardona C, Miles JW, Zuñiga E, Sotelo G. 2010. Independence of resistance in *Brachiaria spp*. to nymphs or to adult spittlebugs (Hemiptera: Cercopidae): implications for breeding for resistance. J Econ Entomol. 103:1860–1865. 10.1603/EC10004. 21061990

[jkag101-B15] Cardona C, Sotelo G. 2005. Mecanismos de resistencia a insectos: naturaleza e importancia en la formulación de estrategias de mejoramiento para incorporar resistencia a salivazo en *Brachiaria*. Pasturas Trop. 27:1–11. https://www.tropicalgrasslands.info/public/journals/4/Elements/DOCUMENTS/2005-vol27-rev1-2-3/Vol_27_rev2_05_pags_2-11.pdf.

[jkag101-B16] Congio GFS et al 2020. Spittlebug damage on tropical grass and its impact in pasture-based beef production systems. Sci Rep. 10:10758. 10.1038/s41598-020-67490-9. 32612122 PMC7329844

[jkag101-B17] Covarrubias-Pazaran GE . 2020. Heritability: meaning and computation. CIMMYT. p. 23.

[jkag101-B18] da Costa Lima Moraes A et al 2023. Advances in genomic characterization of Urochloa humidicola: exploring polyploid inheritance and apomixis. Theor Appl Genet. 136:238. 10.1007/s00122-023-04485-w. 37919432

[jkag101-B19] Deepak S et al 2010. Hydroxyproline-rich glycoproteins and plant defence. J Phytopathol. 158:585–593. 10.1111/j.1439-0434.2010.01669.x.

[jkag101-B20] Demirjian C, Vailleau F, Berthomé R, Roux F. 2023. Genome-wide association studies in plant pathosystems: success or failure? Trends Plant Sci. 28:471–485. 10.1016/j.tplants.2022.11.006. 36522258

[jkag101-B21] do Carmo Santos ML et al 2025. The family of glutathione peroxidase proteins and their role against biotic stress in plants: a systematic review. Front Plant Sci. 16:1425880. 10.3389/fpls.2025.1425880. 40051871 PMC11882536

[jkag101-B22] Espitia-Buitrago P et al 2025. Enhancing phenotyping accuracy for selection of *Urochloa spp*. tolerant genotypes to red spider mite (*Oligonychus trichardti*). Grass Forage Sci. 80:e70007. 10.1111/gfs.70007.

[jkag101-B23] Ferreira RCU et al 2019. Genetic mapping with allele dosage information in tetraploid *Urochloa decumbens* (Stapf) R. D. Webster reveals insights into spittlebug (Notozulia entreriana Berg) resistance. Front Plant Sci. 10:92. 10.3389/fpls.2019.00092. 30873183 PMC6401981

[jkag101-B24] Ferreira RCU et al 2021. An overview of the genetics and genomics of the *Urochloa* species most commonly used in pastures. Front Plant Sci. 12:770461. 10.3389/fpls.2021.770461. 34966402 PMC8710810

[jkag101-B25] Ferreira T, Rasband W. 2012. ImageJ user guide. USA: National Institutes of Health. https://imagej.net/ij/docs/guide/user-guide.pdf.

[jkag101-B26] Forsthoefel NR, Cutler K, Port MD, Yamamoto T, Vernon DM. 2005. PIRLs: a novel class of plant intracellular leucine-rich repeat proteins. Plant Cell Physiol. 46:913–922. 10.1093/pcp/pci097. 15809230

[jkag101-B27] González-Lamothe R et al 2006. The U-box protein CMPG1 is required for efficient activation of defense mechanisms triggered by multiple resistance genes in tobacco and tomato. Plant Cell. 18:1067–1083. 10.1105/tpc.106.040998. 16531490 PMC1425846

[jkag101-B30] Hernández L, Espitia P, Cardoso JA. 2022a. Digital imaging outperforms traditional scoring methods for spittlebug tolerance in *Urochloa humidicola* hybrids. Trop Grassl Forrajes Trop. 10:271–279. 10.17138/TGFT(10)271-279.

[jkag101-B28] Hernández LM et al 2021. Geographic distribution of Colombian spittlebugs (Hemiptera: Cercopidae) via ecological niche modeling: a prediction for the main tropical forages’ pest in the Neotropics. Front Sustain Food Syst. 5:725774. 10.3389/fsufs.2021.725774.

[jkag101-B29] Hernández LM et al 2022b. Estimation of genetic gain for resistance to spittlebugs (Hemiptera: Cercopidae) in the interspecific Urochloa CIAT breeding program using historical data. Tropentag 2022, International Conference on Research on Food Security, Natural Resource Management and Rural Development; Prague, Czech Republic. Czech University of Life Sciences. https://www.tropentag.de/2022/TT22boa.pdf.

[jkag101-B31] Huang F et al 2025. Exploring resistance mechanisms and identifying QTLs for brown planthopper in tropical and subtropical rice (*Oryza sativa* L.) germplasm. Theor Appl Genet. 138:49. 10.1007/s00122-025-04839-6. 39976729

[jkag101-B32] Huang M, Liu X, Zhou Y, Summers RM, Zhang Z. 2019. BLINK: a package for the next level of genome-wide association studies with both individuals and markers in the millions. Gigascience. 8:giy154. 10.1093/gigascience/giy154. 30535326 PMC6365300

[jkag101-B33] Karthik HN, Parmar S, Gawande ND, Sankaranarayanan S. 2025. Multifaceted roles of U-box E3 ligases in plant development. Plant Cell Physiol. 66:1123–1136. 10.1093/pcp/pcaf059. 40445202

[jkag101-B34] Kawano Y, Kaneko-Kawano T, Shimamoto K. 2014. Rho family GTPase-dependent immunity in plants and animals. Front Plant Sci. 5:522. 10.3389/fpls.2014.00522. 25352853 PMC4196510

[jkag101-B35] Kawasaki T, Henmi K, Ono E, Hatakeyama S, Shimamoto KO. 1999. The small GTP-binding protein Rac is a regulator of cell death in plants. Proc Natl Acad Sci U S A. 96:10922–10926. 10.1073/pnas.96.19.10922. 10485927 PMC17984

[jkag101-B36] Koch KG, Chapman K, Louis J, Heng-Moss T, Sarath G. 2016. Plant tolerance: a unique approach to control hemipteran pests. Front Plant Sci. 7:1363. 10.3389/fpls.2016.01363. 27679643 PMC5020058

[jkag101-B37] Lambrecht JA, Downs DM. 2013. Anthranilate phosphoribosyl transferase (TrpD) generates phosphoribosylamine for thiamine synthesis from enamines and phosphoribosyl pyrophosphate. ACS Chem Biol. 8:242–248. 10.1021/cb300364k. 23101964 PMC3549051

[jkag101-B38] Langmead B, Salzberg SL. 2012. Fast gapped-read alignment with bowtie 2. Nat Methods. 9:357–359. 10.1038/nmeth.1923. 22388286 PMC3322381

[jkag101-B39] Lannoo N, Van Damme EJM. 2014. Lectin domains at the frontiers of plant defense. Front Plant Sci. 5:397. 10.3389/fpls.2014.00397. 25165467 PMC4131498

[jkag101-B40] Lee K-A, Cho T-J. 2003. Characterization of a salicylic acid- and pathogen-induced lipase-like gene in Chinese cabbage. J Biochem Mol Biol. 36:433–441. 10.5483/bmbrep.2003.36.5.433. 14536025

[jkag101-B41] Liu X, Huang M, Fan B, Buckler ES, Zhang Z. 2016. Iterative usage of fixed and random effect models for powerful and efficient genome-wide association studies. PLoS Genet. 12:e1005767. 10.1371/journal.pgen.1005767. 26828793 PMC4734661

[jkag101-B42] McDonald SC, Buck J, Li Z. 2022. Automated, image-based disease measurement for phenotyping resistance to soybean frogeye leaf spot. Plant Methods. 18:103. 10.1186/s13007-022-00934-7. 35974392 PMC9382788

[jkag101-B43] Mitchell C, Brennan RM, Graham J, Karley AJ. 2016. Plant defense against herbivorous pests: exploiting resistance and tolerance traits for sustainable crop protection. Front Plant Sci. 7:1132. 10.3389/fpls.2016.01132. 27524994 PMC4965446

[jkag101-B44] Nitthaisong P et al 2019. Pentaploid apomicts by interspecific hybridization between diploid *Urochloa ruziziensis* and tetraploid apomictic *U. decumbens*. Crop Sci. 59:1648–1656. 10.2135/cropsci2019.01.0035.

[jkag101-B45] Njuguna JN et al 2023. Impact of genotype-calling methodologies on genome-wide association and genomic prediction in polyploids. Plant Genome. 16:e20401. 10.1002/tpg2.20401. 37903749 PMC12807030

[jkag101-B46] Pacheco-Coeto R, Cárdenas-Torres L, Hernández-Rosas F, Valente Hidalgo-Contreras J, Aquino-Pérez G. 2019. Callose and reactive oxygen species expressed in sugar cane leaves by mechanical damage of spittlebugs. Rev Mex Cienc Agríc. 10:105–114. 10.29312/remexca.v0i22.1862.

[jkag101-B47] Pamidi LS et al 2025. Characterization of antixenosis and antibiosis resistance to the fall armyworm *Spodoptera frugiperda* (J.E. Smith) in maize. Phytoparasitica. 53:4. 10.1007/s12600-024-01228-5.

[jkag101-B48] Parsa S, Sotelo G, Cardona C. 2011. Characterizing herbivore resistance mechanisms: spittlebugs on *Brachiaria* spp. as an example. J Vis Exp. 52:3047. 10.3791/3047. PMC3197067 21712800

[jkag101-B49] Pascal S et al 2019. *Arabidopsis* CER1-LIKE1 functions in a cuticular very-long-chain alkane-forming complex. Plant Physiol. 179:415–432. 10.1104/pp.18.01075. 30514726 PMC6426428

[jkag101-B50] Patrignani A, Ochsner TE. 2015. Canopeo: a powerful new tool for measuring fractional green canopy cover. Agron J. 107:2312–2320. 10.2134/agronj15.0150.

[jkag101-B51] Perea C et al 2016. Bioinformatic analysis of genotype by sequencing (GBS) data with NGSEP. BMC Genomics. 17:498. 10.1186/s12864-016-2827-7. 27585926 PMC5009557

[jkag101-B52] Perera IY, Hung CY, Moore CD, Stevenson-Paulik J, Boss WF. 2008. Transgenic *Arabidopsis* plants expressing the type 1 inositol 5-phosphatase exhibit increased drought tolerance and altered abscisic acid signaling. Plant Cell. 20:2876–2893. 10.1105/tpc.108.061374. 18849493 PMC2590728

[jkag101-B53] Peterson RKD, Varella AC, Higley LG. 2017. Tolerance: the forgotten child of plant resistance. PeerJ. 5:e3934. 10.7717/peerj.3934. 29062607 PMC5647859

[jkag101-B54] Pilet-Nayel ML et al 2017. Quantitative resistance to plant pathogens in pyramiding strategies for durable crop protection. Front Plant Sci. 8:1838. 10.3389/fpls.2017.01838. 29163575 PMC5664368

[jkag101-B55] Poland JA, Balint-Kurti PJ, Wisser RJ, Pratt RC, Nelson RJ. 2009. Shades of gray: the world of quantitative disease resistance. Trends Plant Sci. 14:21–29. 10.1016/j.tplants.2008.10.006. 19062327

[jkag101-B56] Priyadarshan PM . 2019. Host plant resistance breeding. In: Plant breeding: classical to modern. 1st ed Springer Nature Singapore. p. 379–412. 10.1007/978-981-13-7095-3_18.

[jkag101-B57] Pudjihartono N, Fadason T, Kempa-Liehr AW, O'Sullivan JM. 2022. A review of feature selection methods for machine learning-based disease risk prediction. Front Bioinform. 2:927312. 10.3389/fbinf.2022.927312. 36304293 PMC9580915

[jkag101-B58] Reid JM, Acker P. 2022. Properties of phenotypic plasticity in discrete threshold traits. Evolution. 76:190–206. 10.1111/evo.14408. 34874068

[jkag101-B59] Roberts MR et al 1993. Gametophytic and sporophytic expression of an anther-specific *Arabidopsis thaliana* gene. Plant J. 3:111–120. 10.1046/j.1365-313x.1993.t01-5-00999.x. 8401599

[jkag101-B60] Ryan C et al 2025. A haplotype-resolved chromosome-level genome assembly of *Urochloa decumbens* cv. Basilisk resolves its allopolyploid ancestry and composition. G3 (Bethesda). 15:jkaf005. 10.1093/g3journal/jkaf005. 39854285 PMC12005165

[jkag101-B61] Scully TW, Jiao W, Mittelstädt G, Parker EJ. 2023. Structure, mechanism and inhibition of anthranilate phosphoribosyltransferase. Philos Trans R Soc Lond B Biol Sci. 378:20220039. 10.1098/rstb.2022.0039. 36633281 PMC9835598

[jkag101-B62] Silva SEB et al 2017. Biological performance and preference of *Mahanarva spectabilis* (Hemiptera: Cercopidae) for feeding on different forage plants. J Econ Entomol. 110:1877–1885. 10.1093/jee/tox180. 28854657

[jkag101-B63] Silva SEB et al 2019. Olfactory response of *Mahanarva spectabilis* (Hemiptera: Cercopidae) to volatile organic compounds from forage grasses. Sci Rep. 9:10284. 10.1038/s41598-019-46693-9. 31311958 PMC6635515

[jkag101-B64] Simeoni F, Divita G. 2007. The Dim protein family: from structure to splicing. Cell Mol Life Sci. 64:2079–2089. 10.1007/s00018-007-7043-9. 17558560 PMC11138440

[jkag101-B65] Souza BHS . 2025. Host plant resistance: is it time for a new model?. Neotrop Entomol. 54:82. 10.1007/s13744-025-01300-7. 40637776

[jkag101-B66] Spitzer C et al 2006. The *Arabidopsis elch* mutant reveals functions of an ESCRT component in cytokinesis. Development. 133:4679–4689. 10.1242/dev.02654. 17090720

[jkag101-B67] Stout MJ, Bernaola L, Acevedo F. 2024. Recent history and future trends in host-plant resistance. Ann Entomol Soc Am. 117:139–149. 10.1093/aesa/saae006.

[jkag101-B68] Strandén I, Jenko J. 2024. A computationally feasible multi-trait single-step genomic prediction model with trait-specific marker weights. Genet Sel Evol. 56:58. 10.1186/s12711-024-00926-2. 39152403 PMC11328383

[jkag101-B69] The UniProt Consortium . 2025. UniProt: the universal protein knowledgebase in 2025. Nucleic Acids Res. 53:D609–D617. 10.1093/nar/gkae1010. 39552041 PMC11701636

[jkag101-B70] Urbanek S . 2011. jpeg: Read and write JPEG images. CRAN: Package jpeg - R Project. 10.32614/CRAN.package.jpeg.

[jkag101-B71] VanRaden PM . 2008. Efficient methods to compute genomic predictions. J Dairy Sci. 91:4414–4423. 10.3168/jds.2007-0980. 18946147

[jkag101-B72] Verma S et al 2024. Integrating marker-assisted (MAS) and genomic selection (GS) for plant functional trait improvement. In: Kumar N, Singh H, editors. Plant functional traits for improving productivity. Springer Nature Singapore. p. 203–215.

[jkag101-B901] Vidotti MS et al 2019. Additive and heterozygous (dis) advantage GWAS models reveal candidate genes involved in the genotypic variation of maize hybrids to Azospirillum brasilense. PloS one. 14:e0222788. 31536609 10.1371/journal.pone.0222788 PMC6752820

[jkag101-B73] Wang Y et al 2021. Roles of the 2-oxoglutarate-dependent dioxygenase superfamily in the flavonoid pathway: a review of the functional diversity of F3H, FNS I, FLS, and LDOX/ANS. Molecules. 26:6745. 10.3390/molecules26216745. 34771153 PMC8588099

[jkag101-B74] Wang L, Wu J. 2013. The essential role of jasmonic acid in plant-herbivore interactions: using the wild tobacco *Nicotiana attenuata* as a model. J Genet Genomics. 40:597–606. 10.1016/j.jgg.2013.10.001. 24377866

[jkag101-B75] Wang J, Zhang Z. 2021. GAPIT version 3: boosting power and accuracy for genomic association and prediction. Genom Proteom Bioinf. 19:629–640. 10.1016/j.gpb.2021.08.005. PMC9121400 34492338

[jkag101-B76] Worthington M et al 2016. A parthenogenesis gene candidate and evidence for segmental allopolyploidy in apomictic *Brachiaria decumbens*. Genetics. 203:1117–1132. 10.1534/genetics.116.190314. 27206716 PMC4937464

[jkag101-B77] Worthington M et al 2019. Translocation of a parthenogenesis gene candidate to an alternate carrier chromosome in apomictic *Brachiaria humidicola*. BMC Genomics. 20:41. 10.1186/s12864-018-5392-4. 30642244 PMC6332668

[jkag101-B78] Worthington M et al 2021. A new genome allows the identification of genes associated with natural variation in aluminium tolerance in *Brachiaria* grasses. J Exp Bot. 72:302–319. 10.1093/jxb/eraa469. 33064149 PMC7853602

[jkag101-B79] Worthington ML, Miles JW. 2015. Reciprocal full-sib recurrent selection and tools for accelerating genetic gain in apomictic *Brachiaria*. In: Budak H, Spangenber G, editors. Molecular breeding of forage and turf. Springer International Publishing. p. 111–122. 10.1007/978-3-319-08714-6_10.

[jkag101-B80] Xiao Q, Bai X, Zhang C, He Y. 2022. Advanced high-throughput plant phenotyping techniques for genome-wide association studies: a review. J Adv Res. 35:215–230. 10.1016/j.jare.2021.05.002. 35003802 PMC8721248

[jkag101-B81] Yu F et al 2020. ESCRT-I component VPS23A is targeted by E3 ubiquitin ligase XBAT35 for proteasome-mediated degradation in modulating ABA signaling. Mol Plant. 13:1556–1569. 10.1016/j.molp.2020.09.008. 32919085

[jkag101-B82] Yuan-Yuan L et al 2018. Immunolocalization and changes of hydroxyproline-rich glycoproteins during symbiotic germination of *Dendrobium officinale*. Front Plant Sci. 9:552. 10.3389/fpls.2018.00552. 29922306 PMC5996918

[jkag101-B83] Zechmann B . 2020. Subcellular roles of glutathione in mediating plant defense during biotic stress. Plants. 9:1067. 10.3390/plants9091067. 32825274 PMC7569779

[jkag101-B84] Zhang C, Dong SS, Xu JY, He WM, Yang TL. 2019. PopLDdecay: a fast and effective tool for linkage disequilibrium decay analysis based on variant call format files. Bioinformatics. 35:1786–1788. 10.1093/bioinformatics/bty875. 30321304

[jkag101-B85] Zheng H et al 2022. A novel putative 2-oxoglutarate-dependent dioxygenase gene (BoaAOP-like) regulates aliphatic glucosinolate biosynthesis in Chinese kale. Sci Hortic. 297:110921. 10.1016/j.scienta.2022.110921.

