# Supplementary material for: Integrating image-based phenotyping and GWAS to map resistance to spittlebug nymphs in interspecific Urochloa grasses
Source: G3 (Bethesda). 2026 Apr 27;16(6):jkag101. doi: 10.1093/g3journal/jkag101 (PMC13232496; doi:10.1093/g3journal/jkag101)
Supplement: jkag101_Supplementary_Data [file jkag101_supplementary_data.zip › Supplementary_table_S2_G3-2026-406667.docx]

**Suppl. Table S1.** Cullis broad-sense heritability obtained from the single trial analyses per trial.

| **Trait** | **T1** | **T2** | **T3** | **T4** | **T5** | **T6** | **T7** |
| --- | --- | --- | --- | --- | --- | --- | --- |
| Total plant damage (DQU) | 0.647 | 0.698 | 0.43 | 0.429 | 0.616 | 0.761 | 0.738 |
| Total plant damage (DTR) | 0.54 | 0.01 | 0.462 | 0.447 | 0.577 | 0.821 | 0.664 |
| Yellow tissue (DTR) | 0.52 | 0.119 | 0.534 | 0.751 | 0.372 | 0.824 | 0.999 |
| Necrotic tissue (DTR) | 0.558 | 0 | 0.304 | 0.107 | 0.818 | 0.6 | 0.554 |
| Insect survival (%) | - | 0.045 | 0.622 | - | 0.369 | 0.303 | 0.352 |
