# Supplementary material for: Integrating image-based phenotyping and GWAS to map resistance to spittlebug nymphs in interspecific Urochloa grasses
Source: G3 (Bethesda). 2026 Apr 27;16(6):jkag101. doi: 10.1093/g3journal/jkag101 (PMC13232496; doi:10.1093/g3journal/jkag101)
Supplement: jkag101_Supplementary_Data [file jkag101_supplementary_data.zip › Supplementary_table_S4_G3-2026-406667.docx]

**Suppl. Table S2.** Parameters of significant SNPs associated with plant damage traits in the *Urochloa* interspecific population. NT = Necrotic tissue. TPD = Total Plant Damage.

| **Chr** | **Position** | **Model** | **Estimator** | **Trait** | **MAF** | **PVE** | **Effect** | **P value** | **FDR** | **−log₁₀(P-value)** |
| --- | --- | --- | --- | --- | --- | --- | --- | --- | --- | --- |
| 1 | 11,344,775 | BLINK | BLUP | NT (DTR) | 0.428 | 10.203 | 0.010 | 5.19E-08 | 1.48E-03 | 7.285 |
| 1 | 11,524,270 | FarmCPU | Mean | TPD (DQU) | 0.483 | 10.123 | 0.023 | 3.06E-07 | 4.35E-03 | 6.514 |
| 1 | 11,524,270 | FarmCPU | BLUP | TPD (DQU) | 0.483 | 10.123 | 0.023 | 3.06E-07 | 4.35E-03 | 7.082 |
| 5 | 63,386,517 | FarmCPU | BLUE | TPD (DQU) | 0.188 | 8.919 | -0.034 | 2.56E-07 | 4.85E-03 | 7.044 |
| 6 | 64,524,508 | BLINK | BLUP | NT (DTR) | 0.337 | 5.413 | 0.019 | 8.57E-08 | 1.22E-03 | 6.334 |
| 6 | 64,524,508 | BLINK | Mean | TPD (DQU) | 0.337 | 5.413 | 0.019 | 8.57E-08 | 1.22E-03 | 6.540 |
| 6 | 64,524,508 | BLINK | BLUE | TPD (DQU) | 0.337 | 5.413 | 0.019 | 8.57E-08 | 1.22E-03 | 7.067 |
| 6 | 64,524,508 | BLINK | BLUP | TPD (DQU) | 0.337 | 5.413 | 0.019 | 8.57E-08 | 1.22E-03 | 7.086 |
| 6 | 64,524,508 | FarmCPU | BLUP | TPD (DQU) | 0.337 | 5.413 | 0.019 | 8.57E-08 | 1.22E-03 | 6.592 |
| 6 | 64,524,508 | BLINK | Mean | TPD (DTR) | 0.337 | 5.413 | 0.019 | 8.57E-08 | 1.22E-03 | 8.606 |
| 6 | 64,524,508 | BLINK | BLUE | TPD (DTR) | 0.337 | 5.413 | 0.019 | 8.57E-08 | 1.22E-03 | 7.778 |
| 6 | 64,524,508 | BLINK | BLUP | TPD (DTR) | 0.337 | 5.413 | 0.019 | 8.57E-08 | 1.22E-03 | 8.646 |
| 7 | 28,519,388 | BLINK | BLUP | NT (DTR) | 0.074 | 21.515 | -0.053 | 3.28E-09 | 9.33E-05 | 7.469 |
| 7 | 28,519,388 | FarmCPU | BLUP | NT (DTR) | 0.074 | 21.515 | -0.053 | 3.28E-09 | 9.33E-05 | 6.642 |
| 7 | 28,519,388 | BLINK | Mean | TPD (DQU) | 0.074 | 21.515 | -0.053 | 3.28E-09 | 9.33E-05 | 7.298 |
| 7 | 28,519,388 | BLINK | BLUE | TPD (DQU) | 0.074 | 21.515 | -0.053 | 3.28E-09 | 9.33E-05 | 8.484 |
| 7 | 28,519,388 | BLINK | BLUP | TPD (DQU) | 0.074 | 21.515 | -0.053 | 3.28E-09 | 9.33E-05 | 9.009 |
| 7 | 28,519,388 | FarmCPU | Mean | TPD (DQU) | 0.074 | 21.515 | -0.053 | 3.28E-09 | 9.33E-05 | 7.793 |
| 7 | 28,519,388 | FarmCPU | BLUE | TPD (DQU) | 0.074 | 21.515 | -0.053 | 3.28E-09 | 9.33E-05 | 6.320 |
| 7 | 28,519,388 | FarmCPU | BLUP | TPD (DQU) | 0.074 | 21.515 | -0.053 | 3.28E-09 | 9.33E-05 | 6.674 |
| 7 | 28,519,388 | BLINK | Mean | TPD (DTR) | 0.074 | 21.515 | -0.053 | 3.28E-09 | 9.33E-05 | 9.040 |
| 7 | 28,519,388 | BLINK | BLUE | TPD (DTR) | 0.074 | 21.515 | -0.053 | 3.28E-09 | 9.33E-05 | 7.534 |
| 7 | 28,519,388 | BLINK | BLUP | TPD (DTR) | 0.074 | 21.515 | -0.053 | 3.28E-09 | 9.33E-05 | 8.619 |
| 10 | 58,801,952 | BLINK | Mean | TPD (DTR) | 0.422 | 2.388 | 0.024 | 8.07E-07 | 1.15E-02 | 7.302 |
| 12 | 54,712,901 | BLINK | BLUE | TPD (DTR) | 0.262 | 7.041 | 0.030 | 2.94E-07 | 5.57E-03 | 6.828 |
| 12 | 56,114,516 | BLINK | Mean | TPD (DTR) | 0.248 | 9.862 | 0.030 | 2.79E-07 | 5.29E-03 | 8.177 |
| 12 | 56,384,467 | FarmCPU | BLUE | TPD (DQU) | 0.206 | 10.533 | 0.026 | 4.17E-07 | 5.93E-03 | 6.871 |
| 12 | 82,658,095 | FarmCPU | BLUP | NT (DTR) | 0.145 | 4.827 | 0.013 | 3.71E-07 | 1.05E-02 | 8.222 |
| 13 | 3,093,711 | FarmCPU | BLUP | NT (DTR) | 0.306 | 4.974 | -0.009 | 5.65E-07 | 1.07E-02 | 6.097 |
| 14 | 23,536,073 | FarmCPU | BLUP | TPD (DQU) | 0.255 | 6.750 | -0.017 | 5.08E-07 | 4.81E-03 | 9.310 |
| 24 | 52,848,791 | FarmCPU | Mean | TPD (DQU) | 0.443 | 5.347 | -0.035 | 3.95E-08 | 7.49E-04 | 8.615 |
| 27 | 13,950,992 | BLINK | BLUP | NT (DTR) | 0.423 | 4.805 | -0.009 | 7.95E-07 | 9.09E-03 | 8.755 |
| 27 | 31,345,868 | BLINK | BLUE | NT (DTR) | 0.389 | 5.662 | 0.009 | 8.35E-07 | 1.19E-02 | 6.531 |
| 27 | 31,345,868 | FarmCPU | BLUP | NT (DTR) | 0.389 | 5.662 | 0.009 | 8.35E-07 | 1.19E-02 | 6.093 |
| 29 | 57,331,694 | BLINK | Mean | TPD (DQU) | 0.346 | 3.894 | 0.027 | 2.19E-09 | 9.33E-05 | 6.413 |
| 29 | 57,331,694 | FarmCPU | Mean | TPD (DQU) | 0.346 | 3.894 | 0.027 | 2.19E-09 | 9.33E-05 | 6.555 |
| 29 | 57,331,694 | FarmCPU | BLUE | TPD (DQU) | 0.346 | 3.894 | 0.027 | 2.19E-09 | 9.33E-05 | 6.380 |
| 36 | 15,990,330 | BLINK | BLUE | NT (DTR) | 0.255 | 5.388 | -0.023 | 8.75E-07 | 8.85E-03 | 6.100 |
| 36 | 15,990,330 | BLINK | BLUP | NT (DTR) | 0.255 | 5.388 | -0.023 | 8.75E-07 | 8.85E-03 | 6.078 |
| 36 | 15,990,330 | BLINK | Mean | TPD (DQU) | 0.255 | 5.388 | -0.023 | 8.75E-07 | 8.85E-03 | 6.294 |
| 36 | 15,990,330 | BLINK | BLUE | TPD (DQU) | 0.255 | 5.388 | -0.023 | 8.75E-07 | 8.85E-03 | 6.251 |
| 36 | 15,990,330 | BLINK | BLUP | TPD (DQU) | 0.255 | 5.388 | -0.023 | 8.75E-07 | 8.85E-03 | 7.403 |
| 36 | 15,990,330 | FarmCPU | Mean | TPD (DQU) | 0.255 | 5.388 | -0.023 | 8.75E-07 | 8.85E-03 | 6.431 |
| 36 | 15,990,330 | FarmCPU | BLUP | TPD (DQU) | 0.255 | 5.388 | -0.023 | 8.75E-07 | 8.85E-03 | 6.248 |
| 36 | 15,990,330 | BLINK | BLUP | TPD (DTR) | 0.255 | 5.388 | -0.023 | 8.75E-07 | 8.85E-03 | 7.159 |
| 36 | 37,631,131 | FarmCPU | BLUP | TPD (DQU) | 0.307 | 3.717 | -0.023 | 3.26E-07 | 3.71E-03 | 8.660 |
