# Supplementary material for: Integrating image-based phenotyping and GWAS to map resistance to spittlebug nymphs in interspecific Urochloa grasses
Source: G3 (Bethesda). 2026 Apr 27;16(6):jkag101. doi: 10.1093/g3journal/jkag101 (PMC13232496; doi:10.1093/g3journal/jkag101)
Supplement: jkag101_Supplementary_Data [file jkag101_supplementary_data.zip › Supplementary_table_S5_G3-2026-406667.docx]

**Table S.3.** *Urochloa* annotated genes within ±2 kb, ±5 kb, or ±10 kb of the significant MTAs using *Urochloa decumbens* genome annotations

| ***Urochloa* annotated gene** | **Window** |
| --- | --- |
| URODEC1_LOCUS_100248 | 2 kbp |
| URODEC1_LOCUS_106022 | 2 kbp |
| URODEC1_LOCUS_12866 | 2 kbp |
| URODEC1_LOCUS_12969 | 2 kbp |
| URODEC1_LOCUS_12988 | 2 kbp |
| URODEC1_LOCUS_15310 | 2 kbp |
| URODEC1_LOCUS_22598 | 2 kbp |
| URODEC1_LOCUS_6035 | 2 kbp |
| URODEC1_LOCUS_68004 | 2 kbp |
| URODEC1_LOCUS_92620 | 2 kbp |
| URODEC1_LOCUS_996 | 2 kbp |
| URODEC1_LOCUS_997 | 2 kbp |
| URODEC1_LOCUS_100247 | 5 kbp |
| URODEC1_LOCUS_107771 | 5 kbp |
| URODEC1_LOCUS_107772 | 5 kbp |
| URODEC1_LOCUS_12989 | 5 kbp |
| URODEC1_LOCUS_15765 | 5 kbp |
| URODEC1_LOCUS_995 | 5 kbp |
| URODEC1_LOCUS_998 | 5 kbp |
| URODEC1_LOCUS_100246 | 10 kbp |
| URODEC1_LOCUS_1012 | 10 kbp |
| URODEC1_LOCUS_1013 | 10 kbp |
| URODEC1_LOCUS_1014 | 10 kbp |
| URODEC1_LOCUS_12970 | 10 kbp |
| URODEC1_LOCUS_12987 | 10 kbp |
| URODEC1_LOCUS_12990 | 10 kbp |
| URODEC1_LOCUS_15764 | 10 kbp |
| URODEC1_LOCUS_22599 | 10 kbp |
| URODEC1_LOCUS_6034 | 10 kbp |
| URODEC1_LOCUS_68005 | 10 kbp |
| URODEC1_LOCUS_68381 | 10 kbp |
| URODEC1_LOCUS_993 | 10 kbp |
| URODEC1_LOCUS_994 | 10 kbp |
| URODEC1_LOCUS_999 | 10 kbp |
