# Supplementary figures and images for: Integrating image-based phenotyping and GWAS to map resistance to spittlebug nymphs in interspecific Urochloa grasses
Source: G3 (Bethesda). 2026 Apr 27;16(6):jkag101. doi: 10.1093/g3journal/jkag101 (PMC13232496; doi:10.1093/g3journal/jkag101)

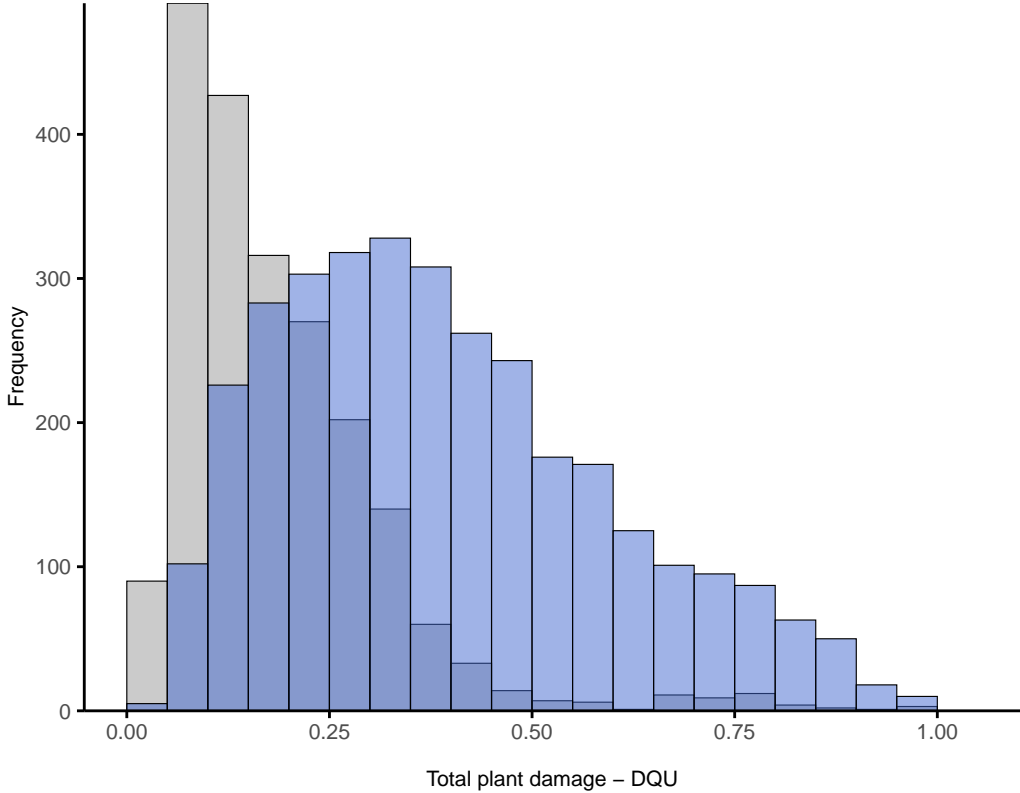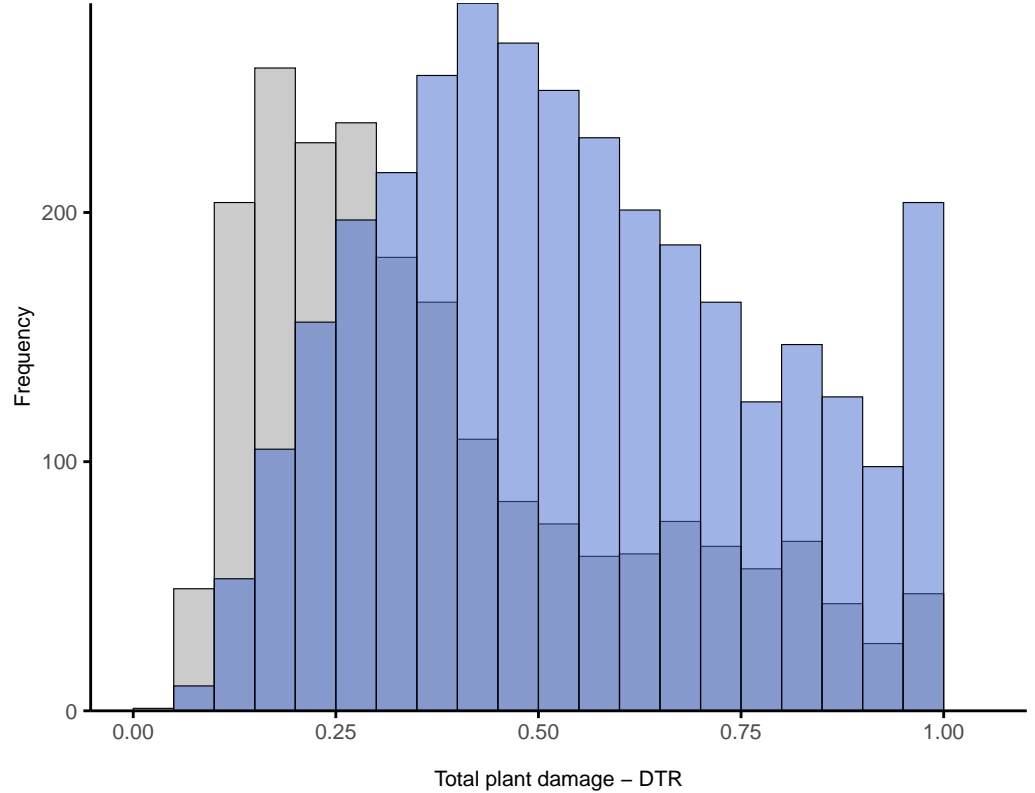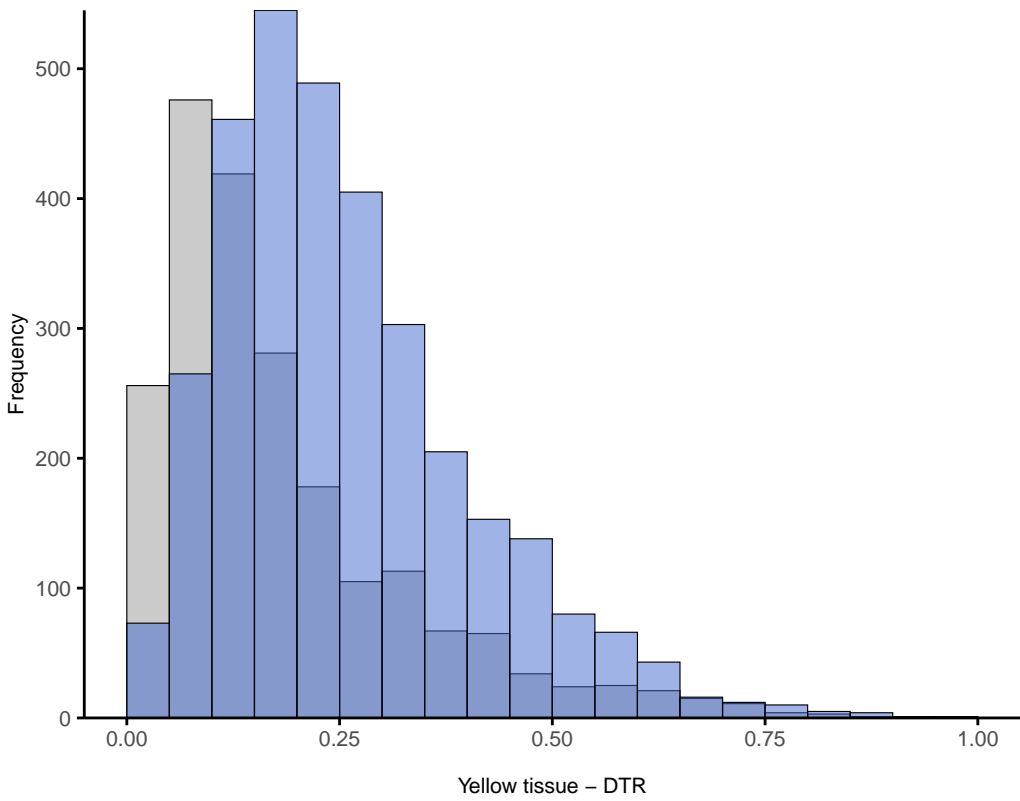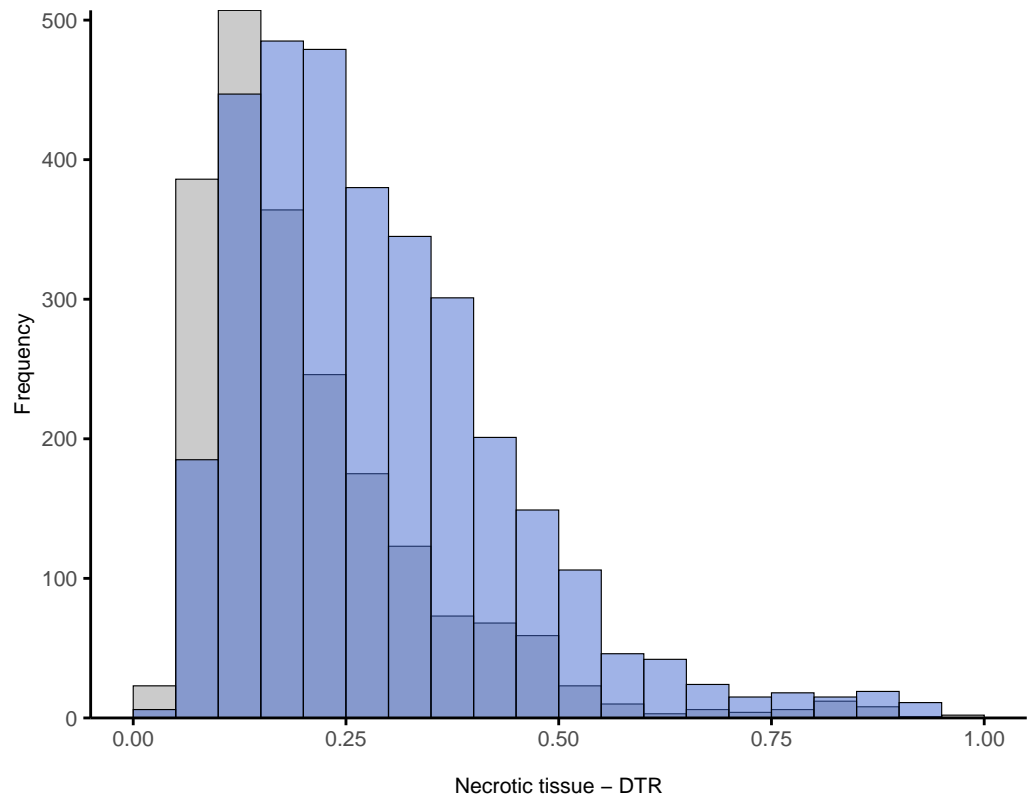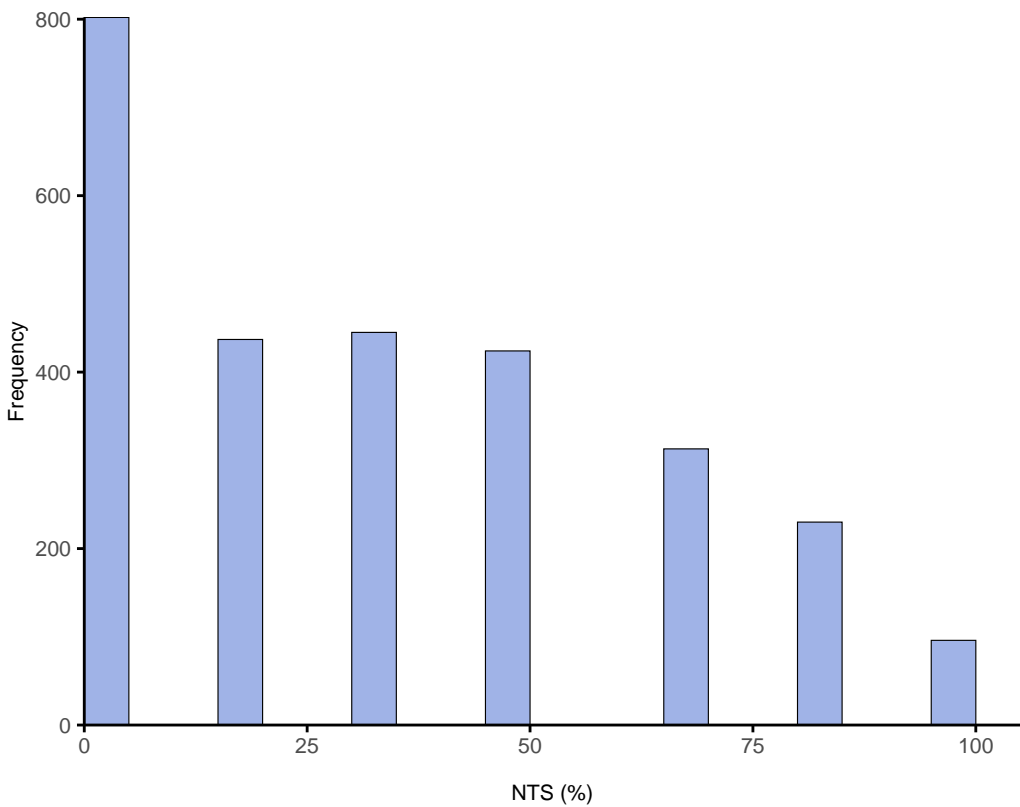

Supplement: jkag101_Supplementary_Data [file jkag101_supplementary_data.zip › Supplemental_Figure_S1_G3-2026-406667.pdf]
